# DNA damage and health effects in juvenile haddock (*Melanogrammus aeglefinus*) exposed to PAHs associated with oil-polluted sediment or produced water

**DOI:** 10.1371/journal.pone.0240307

**Published:** 2020-10-22

**Authors:** Sonnich Meier, Ørjan Karlsen, Jeremie Le Goff, Lisbet Sørensen, Elin Sørhus, Daniela M. Pampanin, Carey E. Donald, Per Gunnar Fjelldal, Evgenia Dunaevskaya, Marta Romano, Ilaria Caliani, Silvia Casini, André S. Bogevik, Pål A. Olsvik, Mark Myers, Bjørn Einar Grøsvik

**Affiliations:** 1 Institute of Marine Research, Bergen, Norway; 2 ADn’tox, Bâtiment Recherche, Centre François Baclesse, Caen, France; 3 SINTEF Ocean AS, Environment and New Resources, Trondheim, Norway; 4 Department of Chemistry Bioscience and Environmental Engineering, Faculty of Science and Technology, University of Stavanger, Stavanger, Norway; 5 NORCE, Randaberg, Norway; 6 Department of Physical, Earth and Environmental Sciences, University of Siena, Siena, Italy; 7 Nofima AS – Norwegian Institute of Food, Fisheries Aquaculture Research, Fyllingsdalen, Norway; 8 Nord Univ, Fac Biosci & Aquaculture, Bodo, Norway; 9 Myers Ecotoxicology Services, LLC, Shoreline, Washington, United States of America; University of Louisville School of Medicine, UNITED STATES

## Abstract

The research objective was to study the presence of DNA damages in haddock exposed to petrogenic or pyrogenic polyaromatic hydrocarbons (PAHs) from different sources: 1) extracts of oil produced water (PW), dominated by 2-ring PAHs; 2) distillation fractions of crude oil (representing oil-based drilling mud), dominated by 3-ring PAHs; 3) heavy pyrogenic PAHs, mixture of 4/5/6-ring PAHs. The biological effect of the different PAH sources was studied by feeding juvenile haddock with low doses of PAHs (0.3–0.7 mg PAH/kg fish/day) for two months, followed by a two-months recovery. In addition to the oral exposure, a group of fish was exposed to 12 single compounds of PAHs (4/5/6-ring) via intraperitoneal injection. The main endpoint was the analysis of hepatic and intestinal DNA adducts. In addition, PAH burden in liver, bile metabolites, gene and protein expression of CYP1A, GST activity, lipid peroxidation, skeletal deformities and histopathology of livers were evaluated. Juvenile haddock responded quickly to both intraperitoneal injection and oral exposure of 4/5/6-ring PAHs. High levels of DNA adducts were detected in livers three days after the dose of the single compound exposure. Fish had also high levels of DNA adducts in liver after being fed with extracts dominated by 2-ring PAHs (a PW exposure scenario) and 3-ring PAHs (simulating an oil exposure scenario). Elevated levels of DNA adducts were observed in the liver of all exposed groups after the 2 months of recovery. High levels of DNA adduct were found also in the intestines of individuals exposed to oil or heavy PAHs, but not in the PW or control groups. This suggests that the intestinal barrier is very important for detoxification of orally exposures of PAHs.

## 1. Introduction

The North Sea is impacted by human activity from many sources, including direct industrial discharges, urban runoff from land, offshore discharges from oil platforms and ship traffic, and atmospheric deposition from long-range transport of pollutants [1]. A major concern for the North Sea environment has been chronic and acute discharges of petroleum compounds, including polyaromatic hydrocarbons (PAHs), from more than 500 oil and gas (O&G) installations. As many of the oil fields move into a late production phase, discharges of produced water (PW, water from the reservoir or water that is injected into well to aid the removal of oil) increase [2]. The Tampen region in the Northern part of the North Sea holds some of the main oil fields both in the Norwegian and British sectors, with extensive production for more than 40 years. Oil pollution in the Tampen region has several possible sources, including PW and large deposits of oil-contaminated drill cuttings, *i*.*e*. solid material removed from bore drilling [3, 4]. Presently, the Tampen region contributes with approx. 60% of the total discharges of PW from the Norwegian offshore oil fields. Beyond oil, other sources of PAHs may be pyrolytic PAHs either from incomplete combustion of flaring from the platforms during well testing or from atmospheric input. Sediments in the North Sea contain a general background level of mainly pyrogenic PAHs [5].

Environmental conditions around oil fields have been monitored since the early 2000s by studying fish and invertebrates using a suit of chemical and biological effect parameters [6–8]. The detection of DNA adducts in wild fish is associated with polyaromatic hydrocarbon (PAH) exposure and is reviewed in Dunn et al. [9] and Varanasi et al. [10]. Balk et al. [7] reported for the first time that wild caught fish in the North Sea were negatively affected by discharges from the offshore oil industry activity. They found that Atlantic haddock (*Melanogrammus aeglefinus*) collected in 2002 at the Tampen region had elevated levels of DNA adducts and altered responses in other biomarkers (e.g. PAH metabolites in bile) compared to fish from reference areas. The presence of DNA adducts in haddock from this region were later confirmed by a series of environmental monitoring studies in 2005, 2008 and 2011 [11–13]. However, while haddock from the O&G production areas had the highest levels of DNA adducts, this monitoring also found that even haddock from the North Sea reference area, Egersund Bank, generally had higher levels of DNA adducts than more pristine area like Iceland or the Barents Sea. This could indicate that the whole North Sea has a general background contamination of PAHs sufficiently high to promote DNA damage in fish [11].

Petrogenic PAHs are dominated by 2- and 3- ring PAHs and have a large contribution of alkylated isomers, while the pyrogenic PAHs are dominated by high molecular PAHs (≥ 4-ring PAHs) and low levels of alkylated PAHs [5]. PW contains mostly 2-ring PAHs (≈90%) and only very low concentration of heavy PAHs. However, during weathering and sedimentation processes the petrogenic PAHs also change their profile in the marine environment towards relatively more heavy PAHs. The profile found in old drill cuttings under platforms from the Tampen region typically contains small amounts of 4- and 5-ring PAHs. Some PAHs are known to be carcinogenic as they are metabolised into reactive intermediates that can bind covalently to the DNA [14]. The formation of PAH-DNA adducts has been connected to the induction of mutations and the development of tumours and cancer and is strongly dependent on the PAH structure and the ability to produce reactive electrophilic metabolites [15–17].

Several studies have shown that the exposure to crude oil and PW can induce DNA adducts in marine fish species, both in the laboratory [18–21] and in field observations after oil spills [22, 23]. Likewise, *in vitro* studies have shown that oils and oil fractions contain genotoxic compounds that induce DNA adducts [24–26]. However, the multitude of genotoxic compounds in crude oil have not yet been identified.

The ^32^P-postlabelling assay is the preferred technique to measure DNA adducts because of its high sensitivity [27]. A major challenge for this technique is that it does not provide structural information for identifying unknown adducts, such as PAHs or other compounds [28]. However, the *patterns* that emerge in the radiograph images of thin-layer chromatography separations can be unique. We hypothesized that unique spot patterns would emerge depending on the PAH or PAH mixture administered; if this hypothesis is supported, then the utility of ^32^P-postlabelling assay could be extended to yield basic source characterization.

The aim of this current study was to investigate the formation of DNA adducts in haddock orally exposed to or injected with different profiles of petrogenic or pyrogenic PAHs. We wanted to understand how different sources of PAHs might induce different responses in DNA damage, in order to aid in the source identification of DNA damage-inducing contaminants in wild haddock caught around oil fields in the North Sea. The main endpoint was the analysis of hepatic and intestinal DNA adducts (using the ^32^P-postlabelling method). Several other endpoints were also analysed, including PAH burden in liver, bile metabolites, gene and protein expression of CYP1A, other biomarkers and histopathology of livers. Several key questions were addressed: does oral exposure to PAHs induce DNA adducts in haddock?; what is the rate of DNA adduct formation during chronic exposure, and how fast will fish recover?; do different PAHs give different DNA adduct patterns from the ^32^P-postlabelling method, and can the “spot position” be used to identify the source of PAH exposure?; how are DNA adduct levels related with other biomarkers responses?

## 2. Material and methods

### 2.1 Chemicals

PAHs; Fluoranthene (FL), Pyrene (PY), Benz[a]anthracene (BAA), Chrysene (CHR), Benzo[b]fluoranthene (BBF), Benzo[k]fluoranthene (BKF), Benzo[e]pyrene (BEP), Benzo[a]pyrene (BAP), Perylene (PER), Dibenz[a,h]anthracene (DBA), Indeno(1,2,3-cd)pyrene (IND), Benzo[ghi]perylene (BP) were all obtained from Chiron (Trondheim, Norway). For the food mixture, the PAH mixture was dissolved in fish oil (NOFIMA, Bergen, Norway). For the injection experiment, PAHs were dissolved in dimethyl sulfoxide (DMSO) (Sigma-Aldrich, Oslo, Norway) to a concentration of 4 mg/ml.

### 2.2 Experimental design

Juvenile haddock were exposed to different PAH mixtures through feed or injection. All fish (total of 525 haddock, 147± 31g) were individually tagged with a passive induced transponder (Trovan pit tag; ID100, Stavanger Norway) and distributed in circular tanks (3 m diameter, 7 m^3^). All animal experiments within the study were approved by NARA, the governmental Norwegian Animal Research Authority (http://www.fdu.no/fdu/, reference number FOTS ID 5924).

#### 2.2.1 Exposures through feed experiment

Fish were fed with four different diets (control and three treatments) with automatic feeders 5 times a week with a ratio corresponding to 10 g pellets/kg fish/day (1% of body mass per day). The three treatments are hereafter referred to as *PW*, *Oil*, and *PAH*. Daily PAH doses for the groups were as follows: *PW* treatment group: 0.31 mg PAH/kg fish; *Oil* treatment group: 0.45 mg PAH/kg fish; and *PAH* treatment group: 0.65 mg PAH/kg fish. The *PW* and *Oil* treatment groups contained more than just PAHs, however, and the approximate daily dose of total oil for these treatments was 20 mg oil/kg fish. Details on the preparation of the dosed mixtures and feed are given in section 2.4.

The exposure lasted for 67 days (14^th^ of February–22^nd^ April). At the beginning of the study, there were 105 fish in each treatment tank. At the end of the exposure, approximately 45 fish from each treatment group were retained and fed an uncontaminated diet for a 58 day recovery period until the 19^th^ of June.

The feed composition was modified after 10 days of exposure when we observed that fish from the exposure groups had lower appetite than the control fish. After 2 and 10 days, less food was found in the stomachs of these fish and many fish had not increased their weight. This was believed to be a result of the reduced appetite, since most likely the fish sensed the oil compounds. To reduce the oil compound “smell,” pellets were mixed with a paste made of homogenized prawns before each feeding. This had an immediate positive effect on the appetite of all exposure groups and, from the daily observations of feeding, all groups were eating pellets after the use of prawn paste.

#### 2.2.2 Exposures via injection experiment

Twenty-six haddock were injected with single PAH compounds (2 fish for each of the 12 heavy 4-, 5- and 6-ring PAHs used in the PAH mixture, and 2 control fish) and sampled after three days. This experiment occurred at the same time as the feeding experiment, from exposure day 0 to 3. PAHs were dissolved in DMSO and fish oil to a concentration of 4 mg/ml and each fish was injected in the abdominal cavity with 1 μl/g fish corresponding to a dose of 4 mg/kg fish. Fish were anesthetized before injection (60 mg/l tricaine methanesulfonate, MS 222, Sigma-Aldrich).

### 2.3 Tissue sampling

Four samplings of fish were performed during the feed exposure, and two samplings occurred in the recovery period. The first sampling was three days after first feeding, where the fish had received only one day’s feeding dose. Thereafter followed three samplings at day 10, day 37 and day 67 which was the final day of exposure. Two samplings were performed after exposures ended: at day 7 and at day 58 of the recovery periods. Fish were not fed the day before a sampling in order to be able to collect the bile samples. The fish were killed before sampling by using a high dose of anesthetic (tricaine methanesulfonate, MS 222, Sigma-Aldrich) before sampling the following tissues: bile, plasma, liver, intestine, brain, heart and muscle. Intestine samples were taken from three different positions: the pyloric caeca, the proximal intestine and the distal intestine. All samples were immediately frozen in liquid nitrogen and stored at– 80°C until further analysis. Fish from the injection study were sampled three days after injection and the same tissue samples were taken as in the feed experiment.

### 2.4 Fish feed containing experimental oil mixtures

Three different exposure mixtures of PAH isomers were prepared using three sources: an extract of PW from Statfjord A (Norwegian North Sea, Tampen area), distillation fractions of Gullfaks oil (Norwegian North Sea, Tampen area), and individual commercially purchased PAH standards. The distillation fractions of the Gullfaks oil contain the oil components separated by their boiling points: Fr1 (240–320 °C); Fr2 (320–375°C); Fr3 (375–400 °C). *PW* and *Oil* contained a natural high complexity of PAHs and other oil components, while *PAH* is an artificially created mixture. The PAH content of each exposure mixture and feed was analysed before the exposures began (See section 3.1).

The mixture used in the *PW* treatment was made primarily with an extract of PW. A large volume of PW was sent with a boat from the platform to NORCE in Stavanger, and extracted in a 1000 L polyethylene tank, with 2x20 L cyclohexane. The extraction was done by mixing the cyclohexane and PW overnight with an electric, stainless steel propeller. The combined 40 L of cyclohexane were reduced to 400 ml with rotavaporer (88 °C). The tank material, polyethylene, may have resulted in some PAH losses, however the PAH content in PW and *PW* treatment were well-characterized (see section 3.1). Analysis revealed that large amounts of the volatile naphthalene were removed from the PW extract while evaporating to concentrate the extract procedure. To compensate for that loss, PW extracts were spiked by adding the Fr1 distillate fraction of the Gullfaks oil, which contains the naphthalene fraction of the oil. The *PW* mixture contained 6 g of Fr1 in 60 g PW extract. From this mixture, 28 g was dissolved into fish oil to a total weight of 200 g.

The *Oil* mixture contained 30 g of Fr2, 36 g of Fr3, and 40 mg pyrene standard. From this mixture, 27 g was dissolved into fish oil to a total weight of 200 g.

The final mixture, *PAH*, contained 34–212 mg (S3 Table in S1 File) of 12 heavy PAHs (4/5/6 rings) dissolved in 50 g of acetone, and thereafter dissolved into fish oil to a total weight of 214 g. These heavy, 4/5/6-ring PAHs represent a pyrogenic PAH signature. Acetone was used to assist solubility of the heavy PAHs and evaporated when producing the feed.

Feed with a high inclusion of fishmeal (68%), and no addition of oil, was produced at Nofima Feed Technology Centre (Bergen, Norway) in a co-rotating twin screw extruder (TX 52, Wenger Manufacturing Inc., Sabetha, KS, USA) with a 3 mm size die, and thereafter dried in a dual layer carousel dryer (Model 200.2; Paul Klockner GmbH, Nistertal, Germany). The feed was split into batches and oil-coated with fish oil and the respective treatment mixtures in a Pegasus vacuum coater (PG-10 VC Lab, Dinnissen BV, Sevenum, Netherlands) (Table 1).

**Table 1:** Composition of the experimental feed.

Diet	Composition	Control	*PW*	*Oil*	*PAH*
	%	kg	kg	kg	kg
Fishmeal^1^	68	9	9	9	9
Wheat^2^	12	1.56	1.56	1.56	1.56
Wheat gluten^3^	10	1.3	1.3	1.3	1.3
Fish-oil^1^	7.5	0.975	0.947	0.948	0.975
PW mixture	-	-	0.028	-	-
Oil mixture	-	-	-	0.027	-
PAH mixture	-	-	-	-	0.001
Vitamin mix^4^	2	0.26	0.26	0.26	0.26
Mineral mix^4^	0.52	0.0676	0.0676	0.0676	0.0676
	100%	13	13	13	13

^1^ Norse-LT, provided by Nordsildmel AS, Norway.

^2^ Provided by Norgesmøllene, Norway.

^3^ Amytex 100 vital, Provided by Tereos Syral, France.

^4^ Provided by Vilomix, Norway.

### 2.5 DNA adduct analysis (32P- postlabelling)

A detailed description of the ^32^P-postlabelling method used to analyse liver and intestine samples is given in Pampanin et al. [28] and Le Goff et al. [29]. Samples of the liver and intestine were homogenized, and the DNA from approximately 100 mg of tissue was extracted by liquid-liquid extraction using phenol/chloroform and precipitation with ethanol. Before extraction, the cell nuclei were isolated using a sucrose gradient and then treated with RNases A and T1. For each extracted sample, the DNA concentration in solution was quantified by its absorbance at 260 nm. Absorbance ratios A260/A280 and A260/A230 were used to verify the quality of DNA solutions (i.e. the absence of contamination by RNA and/or proteins). This protocol for adduct analyses is suitable for the quantification of so-called “bulky” DNA adducts. After enzymatic digestion of the DNA, DNA adducts were labeled with ^32^P-adenosine triphosphate, separated on thin-layer chromatography, and quantitatively compared to the labelling of a known quantity of nucleotides. Results are expressed as the relative amount of adducted nucleotides present in 10^9^ non-labelled nucleotides (nmol adduct/mol normal DNA).

### 2.6 Analysis of PAH metabolites in bile (fix wavelength fluorescence method, FF)

PAH metabolite analysis was performed on bile samples, by first diluting 1:1600 in methanol:water (1:1) [30]. All bile samples were analysed by FF at the wavelength pairs 290/335, 341/383 and 380/430 nm, optimised for the detection of 2- and 3-ring, 4-ring and 5-ring PAH metabolites, respectively. Slit widths were set at 2.5 nm for both excitation and emission wavelengths, and samples were analysed in a quartz cuvette. The fluorescence signal was transformed into pyrene fluorescence equivalents through a standard curve made by pyrene (Sigma St Louis, USA). Pyrene was measured at the same fluorimeter, with the same cuvette, same solvent, and with the same slit settings as the bile samples. It was, however, measured at the optimal wavelength pair of pyrene, 332/374 nm (excitation/emission). The concentration of PAH metabolites in bile samples was expressed as μg pyrene fluorescence equivalents (PFE)/mL bile.

### 2.7 Analysis of PAH in the fish feed and haddock liver (GC-MS/MS)

Extraction of fish pellets and haddock liver samples (0.4–0.5 g) was performed as described in Sørensen et al. [31]. To remove co-extracted lipids, a two-step purification protocol was applied. For the removal of triacylglycerids and phospholipids, gel permeation chromatography was applied according to Sørensen et al. [32], followed by solid phase extraction to remove smaller polar lipids [31]. GC-MS/MS analysis of PAHs and alkyl PAHs was performed according to the method described by Sørensen et al. [33].

### 2.8 Detection of gene expression (qPCR)

Quantitative PCR assay was used to evaluate the expression of several biomarker genes. Selected genes were *cyp1a* (Cytochrome P450 1, alpha), *ahrr* (aryl hydrocarbon receptor repressor), *gadd45a* (growth arrest and DNA-damage-inducible, alpha), *gadd45g* (growth arrest and DNA-damage-inducible, gamma), and *p53* (tumor protein P53, which may be activated due to DNA damage after PAHs exposure). Mean normalized expression (MNE) of the target genes was determined using a normalization factor based upon *actb* and *uba52*, calculated by the QuantStudio^™^ Design & Analysis Software. Details about the PCR primers and probes, contig names, amplicon sizes, analysis system and instruments are provided in S5 Table in S1 File.

#### 2.8.1 Detection of *cyp1a* expression

Total RNA was isolated from the homogenized liver and intestines samples using Trizol reagent (Invitrogen, Carlsbad, California, USA) according to procedure provided by the manufacturer. The DNase treatment step was performed using a TURBO DNA-*free* kit (Life Technologies Corporation). Total RNA was quantitated using a NanoDrop^™^-1000 spectrophotometer (Thermo Scientific) and quality was checked on randomly selected samples using Agilent 2100 bioanalyzer (Agilent technologies). cDNA was generated SuperScript VILO cDNA synthesis kit (Thermo Fisher Scientific).

Real time qPCR was performed referring to Taqman Fast advanced Master Mix Protocol (*cyp1a)* (Thermo Fisher Scientific). The plate was read using QuantStudio 5 Real-Time PCR System. For detailed overview see S5 Table in S1 File. Gene expression data for *cyp1a* was calculated relative to the control sample at the same sampling time point using the ΔΔCt method as described in detail in Bogerd et. al (2001).

#### 2.8.2 Detection of *ahrr*, *gadd45a*, *gadd45* and *p53* expression

See S4 Section in S1 File.

### 2.9 Other biomarkers: CYP1A protein in liver (ELISA), Glutathione S-Transferase (GST) activity and lipid peroxidation (LPO)

Enzyme-linked immunosorbent assay (ELISA) was performed as described in Nilsen et al. [34] to determine levels of CYP1A proteins in 0.5 g liver samples. First, total protein concentrations were determined. The liver samples were homogenized in a buffer with a Potter Elvehjem homogenizer (7 strokes). After centrifugation, the (postmitochondrial) supernatant was diluted 1:1000 in dH_2_O. Aliquots were added to a Nunc 96-well plate in triplicate and incubated 5 minutes in Coomassie G-250 / 17% phosphoric acid (1:1), and protein content was measured by absorbance at 595 nm by plate reader (Tecan SPECTRA Fluor). Protein concentration was determined by standard curve with bovine serum albumin. Then, for the ELISA analysis, sample aliquots containing 1 μg total protein were added on two plates with four replicates for each. For measurements of CYP1A1 in liver we used monoclonal mouse antibody (anti-cod CYP1A, NP-7, Biosense, Norway), diluted 1:1000. For secondary antibodies, we used polyclonal goat anti-mouse/rabbit from DacoCytomation (Denmark) diluted 1:2000. Plates were incubated with TMB substrate for 22.5 minutes before addition of 0.5 M H_2_SO_4_ and absorbance read at 450 nm.

Two additional biomarkers were measured in liver samples: GST activity as an indicator of detoxification pathways, and LPO as a measure of oxidative stress. Methods are provided in Supplementary Information.

### 2.10 Liver damage and histopathology

Liver samples were examined macroscopically for health parameters related to physiological conditions, inflammatory and non-specific pathologies and those associated with pathogen and parasites infections. The liver tissue was then dissected, put in a histocassette and placed into the fixative (3.7% formaldehyde) for wax sections. Tissue samples were no thicker than 1 cm to ensure proper fixation. Histological sections (3 μm) were prepared at Helse Stavanger (HS), and blindly analyzed twice: at NORCE (Norway) and by Mark Myers (Seattle, USA). All micrographs were captured using an AxioCam MRc5 (Zeiss) digital camera mounted on a *Zeiss Axioplan 2* light microscope (Göttingen, Germany). The slides were analysed blind.

### 2.11 X-rays of the vertebral column

Whole fish were radiographed laterally with a Computed Radiography (CR) system (CR 35 VET; Dürr Medical, Bietigheim- Bissingen, Germany) using a portable xray unit (Portable X-ray Unit Hiray Plus, Model Porta 100 HF, JOB Corporation, Yokohama, Japan) at 70 cm distance with 40 kV and 10 mAs. Fish with more than one deformed vertebra were classified as deformed.

### 2.12 Statistics

Statistical analyses were performed using XLSTAT software (Addinsoft, US). One-way ANOVA and Dunnett’s post-hoc tests were used to analyse statistical differences in most variables (except DNA adducts). For the DNA adduct measurement large variations were observed between the treatment groups. In the control group, majorities samples were under the detection limit of the method (35 out of 54 samples = 65%). DNA adduct distribution were tested with Fishers Exact Test. For the samples with measurements above detection limits, statistical differences between the treatment groups was analysed using nonparametric tests; Kruskal-Wallis Test and Dunn multiple pairwise comparison.

### 2.13 Ethics statement

All animal experiments within the study were approved by NARA, the governmental Norwegian Animal Research Authority (http://www.fdu.no/fdu/, reference number FOTS ID 5924). All methods were performed in accordance with approved guidelines. All fish were killed before sampling with a high dose MS-222. The animals were monitored daily, and dead fish were removed. There was low mortality during the experiment; in total, 19 fish died (<5%), and mortality was not correlated to exposure treatments.

The Austevoll Research station has the following permission for catch and maintenance of Atlantic haddock: H-AV 77, H-AV 78 and H-AV 79. These are permits given by the Norwegian Directorate of Fisheries. Furthermore, the Austevoll Research station has a permit to run as a Research Animal facility using fish (all developmental stages), with code 93 from the National IACUC; NARA.

## 3. Results

Our results indicate that juvenile haddock responded quickly to both oral exposure and intraperitoneal injection of PAHs. PAHs in the three feed treatments were each distinct in their profiles based on the sizes of the predominating PAHs. Reduced weight compared to control was observed in all oral-exposed groups. Elevated levels of DNA adducts were observed in all of the oral-exposed groups (*PW*, *Oil* and *PAH*), and for 9 of the 12 PAHs administered in the injection experiment. Responses of DNA adducts were initially different among treatments but became more similar over time. Patterns on the radiograms of the DNA adduct assay were inadequate for use as an exposure sourcing technique. Evidence of metabolism was seen in *Oil* and *PAH*, through increased CYP activity and levels of bile metabolites. Concentrations of un-metabolised PAHs were highest in *PW*. In different locations of the intestine, we observed unique DNA adduct and *cyp1a* induction patterns by treatment. Liver damage was recorded but improved after a recovery period, and increased vertebral malformations were observed across oral-exposed treatments. More details are provided in following sections.

### 3.1 PAHs given in the feed experiment

**Fig 1:**
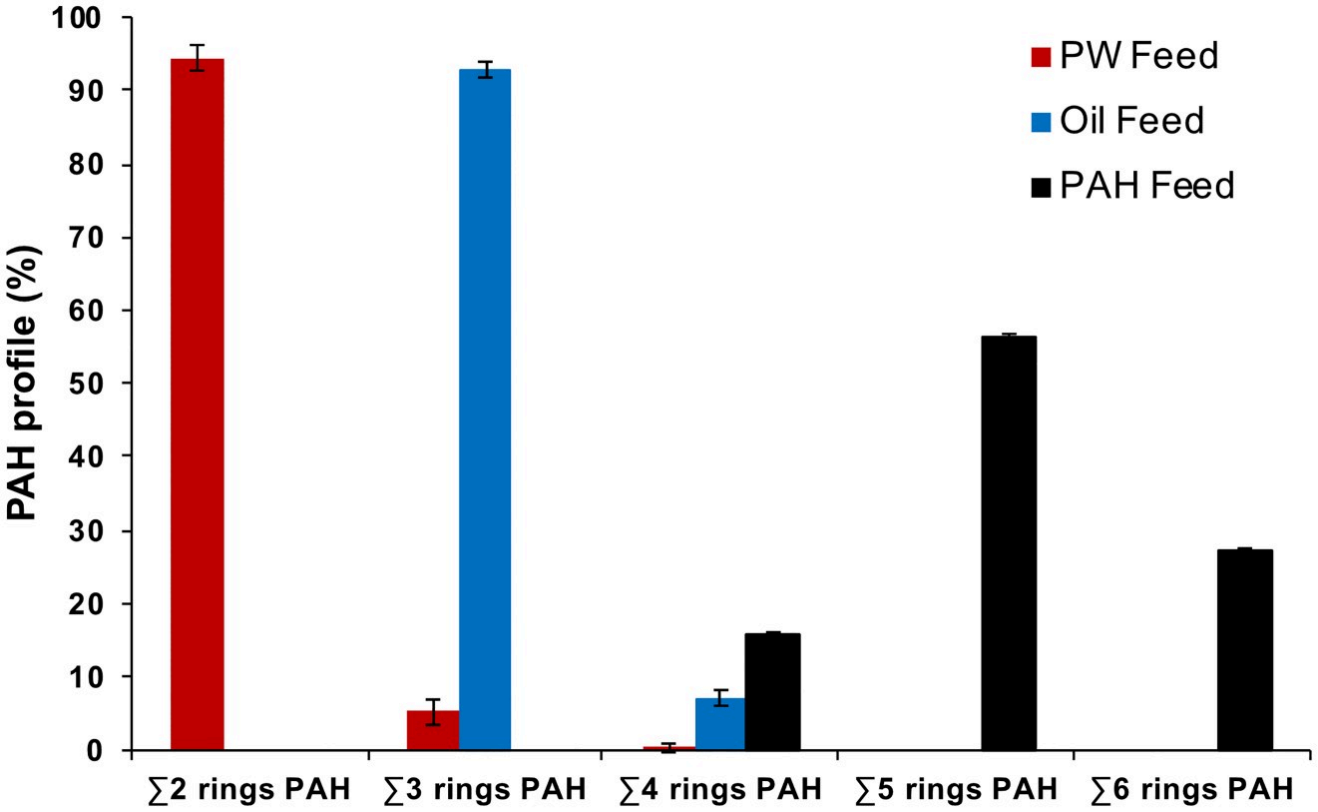
PAH profiles (% of total PAH) in the three exposure feeds; Produced Water (*PW*), oil fraction (*oil*) and heavy *PAH* mixture (n = 3).

Chemical analysis confirmed that the feed experiment included diets with for 3 different profiles of PAHs: *PW* with chiefly 2-ring PAHs; *Oil* with chiefly 3-ring PAHs; and *PAH* with only 4/5/6-ring PAHs (Fig 1). PAHs were the only contaminants that were quantitatively measured in the feed. Measured PAHs contributed 1.1% (*PW*) and 1.5% (*Oil*) of the feed, by mass (Table 2 and S4 Table in S1 File). Therefore, *PW* and *Oil* treatments also contained a complex mixture of other oil compounds, which we characterized using total hydrocarbon analysis gas chromatography-flame ionization detector (GC-FID). Chromatograms of the PW extract and oil distillation fractions show an unresolved complex matrix (UCM, S2 Fig in S1 File).

**Table 2:** Composition of the three PAH sources used in the feed.

	Composition of mixture	Composition in the feed	PAH content (mg PAH/kg feed)	PAH content (%)
***PW***	60 g PW extract + 6 g oil Fr1	28 g of PW mixture in 13 kg feed	31	1.1
***Oil***	30 g oil Fr2 + 36 g oil Fr3 + 0.04 g pyrene	27 g oil mixture in 13 kg feed	45	1.5
***PAH***	12 PAH standards (each 34–212 mg)	976 mg PAH in 13 kg feed	65	100

The PAH content was measured by GC-MS and the PAH profiles are given in S2 Table in S1 File and shown in Fig 1.

### 3.2 Growth measurements in the feed experiment

After 67 days exposure and 58 days of recovery, all the three exposed groups had lower weight compared with control (Table 3). Fish in the *PAH* treatment had the lowest average final weight, length, liver weight, and growth rate—both at the end of exposures (day 67), and after 58 days recovery. Average fish weight for all treatments had increased from starting weights by the 35 day sampling. Control fish weighed 141±28 g at the start of the feed experiment (day 0), and at the end of the recovery period, mean weight was 336±57 g, corresponding to a growth rate of 1.1% day^-1^. The *PAH* exposure, however, had significantly lower weight and growth rate at the end of 67 exposure days (0.4% day^-1^); the rate slightly improved in the recovery period (up to 0.7% day^-1^). The *Oil* treatment occasionally had average weight or growth that was significantly lower than control. Meanwhile, the *PW* treatment’s growth measurements were not different from control until the final sampling at the end of the recovery period.

**Table 3:** Length, weight, liver weight, hepatosomatic index (HSI)^a^; condition factor^b^ and daily growth rate (average daily weight increase since exposure start 14.02) for all treatment groups.

Treatments groups	(n)	Length (mm)	Weight (g)	Liver weight (g)	HSI	Condition factor	Daily growth rate (%)
3 days exposure							
Control	10	225±18	141±28	22±7	15±2	1.23±0.2	-0.2±1.3
*PW*	10	228±12	164±37	25±9	15±3	1.36±0.1	-0.1±0.7
*Oil*	10	230±8	167±32	29±6*	17±1*	1.35±0.1	-1.2±1.5
*PAH*	10	226±7	147±15	20±4	14±1	1.26±0.05	-0.2±07
10 days exposure							
Control	10	230±19	140±31	21±6	15±2	1.15±0.2	0.6±1.4
*PW*	10	228±13	154±33	24±7	16±2	1.29±0.1	0.0±0.2
*Oil*	10	235±12	162±31	28±7*	17±2*	1.23±0.1	-0.2±0.3
*PAH*	10	229±15	166±35	29±7*	18±2*	1.40±0.4	-0.7±03*
37 days (5 weeks) exposure							
Control	15	242±18	192±46	33±9	17±2	1.35±0.1	0.8±0.4
*PW*	15	244±21	201±52	37±13	18±3	1.36±0.1	0.7±0.2
*Oil*	15	241±13	186±41	34±9	18±3	1.31±0.1	0.6±0.2*
*PAH*	15	245±15	192±41	34±9	18±2	1.29±0.1	0.4±0.1*
67 days (9 weeks) exposure							
Control	20	260±13	233±43	41±9	17±2	1.32±0.1	1.0±0.4
*PW*	20	261±16	248±50	45±11	18±2	1.38±0.1	0.8±0.2
*Oil*	20	251±19	206±65	36±17	17±4	1.26±0.2	0.8±0.3
*PAH*	20	247±18*	183±46*	34±9*	19±4	1.19±0.1*	0.4±03*
7 days (1 week) recovery							
Control	10	268±17	265±59	48±13	18±3	1.36±0.1	0.9±0.3
*PW*	10	252±16	235±62	39±15	16±3	1.40±0.1	1.0±0.3
*Oil*	10	261±15	254±42	42±7	17±2	1.45±0.3	0.7±0.2
*PAH*	10	256±13	205±39*	32±10*	16±3	1.21±0.1*	0.4±02*
58 days (9 weeks) recovery							
Control	36	289±17	336±57	62±14	18±3	1.39±0.2	1.1±0.4
*PW*	35	281±21	299±85*	51±19*	17±2*	1.31±0.1	1.1±0.5
*Oil*	31	278±20*	292±75*	51±15*	17±3	1.33±0.1	0.8±0.5*
*PAH*	33	263±21*	236±69*	41±16*	16±5	1.26±0.2*	0.7±0.5*

^a^ $\text{HSI}\;(\%)=\frac{LW}{W}x100$, where LW is the liver weight (g) and *W* is the wet weight of the fish (g).

^b$\text{Condition}\;\text{factior}=\frac{W}{L3}\;x\;100$^, where *W* is the wet weight of the fish (g) and *L* is the length of the fish (cm).

The data are shown as means (±SD). Asterisks indicate statistical difference to the control group. P<0.05.

### 3.3 DNA adducts in liver

#### 3.3.1 DNA adducts in the liver of haddock injected with individual, heavy PAHs

**Fig 2:**
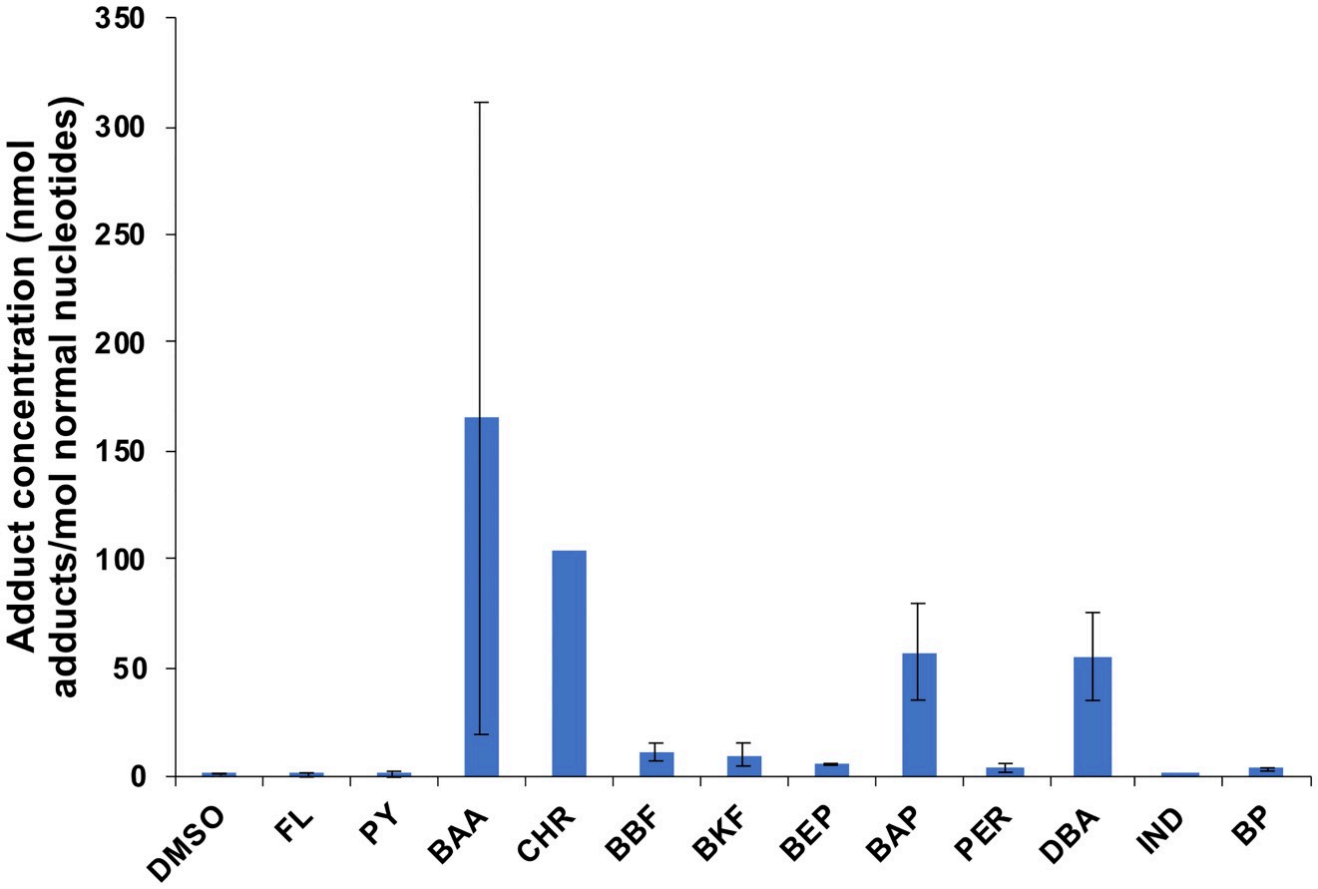
DNA adducts in the liver of fish injected with heavy PAH (4/5/6 rings) (2 replicates of each compound, except chrysene (CHR), where one of the replicates died (n = 1)). Data presented as average ± standard deviation. Dimethylsulfoxide (DMSO) represents the control.

The ^32^P-postlabelling analysis of fish injected with a high dose (4 mg/kg body burden) of a single compound of heavy PAHs showed that some, but not all, PAHs induced DNA adducts (Fig 2). Four compounds had very high levels of DNA adducts (BAA, BAP, CHR, DBA). Three compounds did not induce DNA adducts (FL, PY, IND), and five compounds had detectable but low levels of DNA adducts (BEP, BBF, BKF, PER, BP). The highest measured DNA adducts levels were found in BAA and CHR, with 268 and 105 nmol DNA adduct/mol normal DNA, respectively. There was large variation in intensity between the two replicates for several compounds (RSD from 6–130%), but the pattern regarding none, low or high formation of DNA adducts was consistent (Fig 2 and S3 Fig in S1 File). The results from the injected fish showed that haddock responded quickly to the intraperitoneal injection of PAHs and high amounts of DNA adducts were detected 3 days after injection.

#### 3.3.2 DNA adducts in the liver of haddock exposed through feed

**Fig 3:**
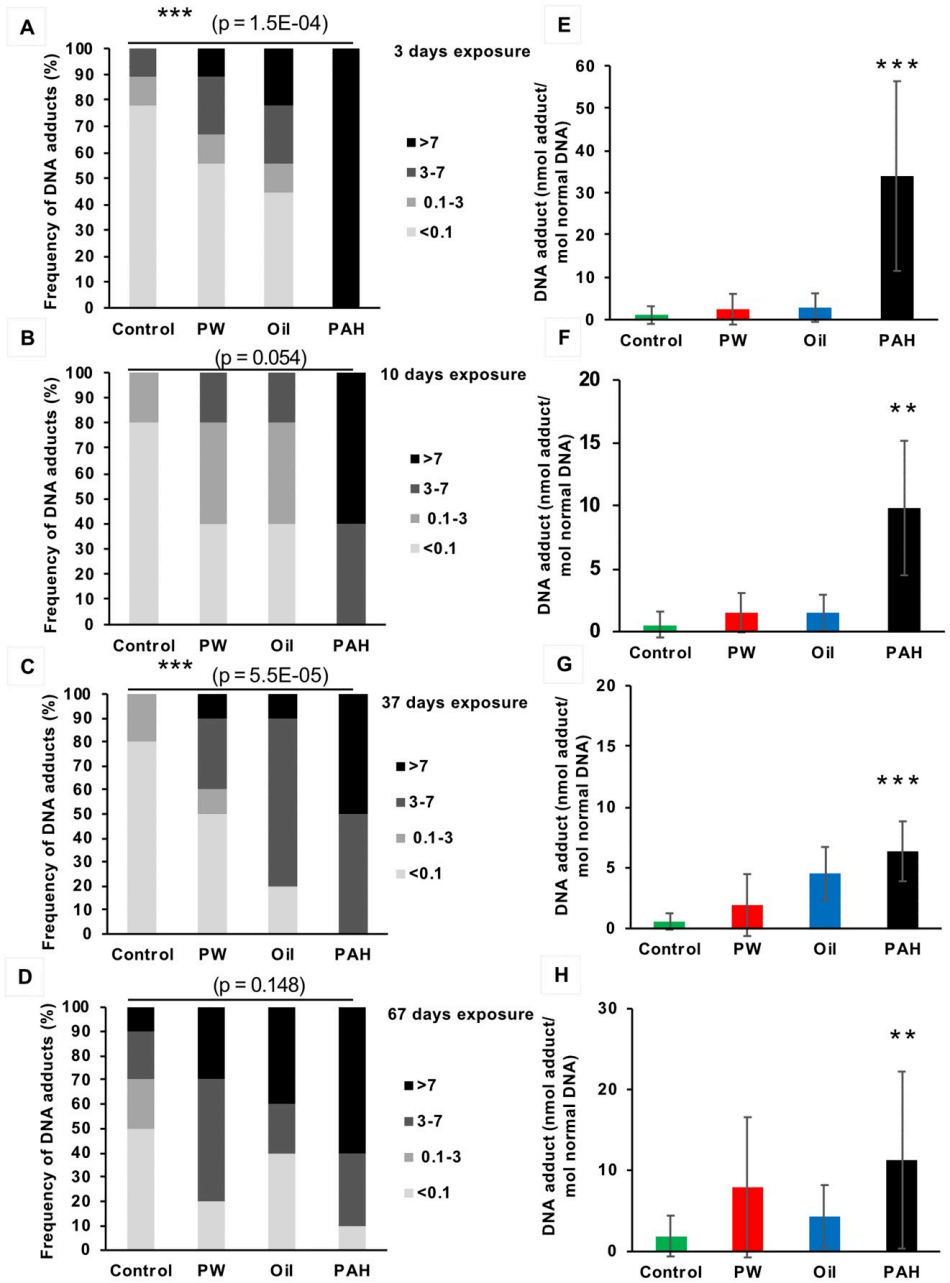
DNA adducts in the liver of fish from the different treatments groups after 3 days (one dose) (A and E, n = 9), 10 days (B and F, n = 5), 37 days (C and G, n = 10) and 67 days (D and H, n = 10) of oral exposure to three exposure feeds; produced water (PW), oil fraction (oil) and heavy PAH mixture and a clean control feed. A-D show the frequency (%) of DNA-adduct divided into four intervals (below detection limit; <0.1 nmol DNA adduct/mol normal DNA, 0.1–3 nmol DNA adduct/mol normal DNA, 3–7 nmol DNA adduct/mol normal DNA, >7 nmol DNA adduct/mol normal DNA. Asterisks indicate statistical difference in the relative distribution between the treatment groups (Fisher Exact Contingency Table analysis). P<0.001 = ***. E-H show the average amount of DNA adducts (±SD). Asterisks indicate statistical difference to the control fish (Kruskal-Wallis Test). P<0.01 = **.

**Fig 4:**
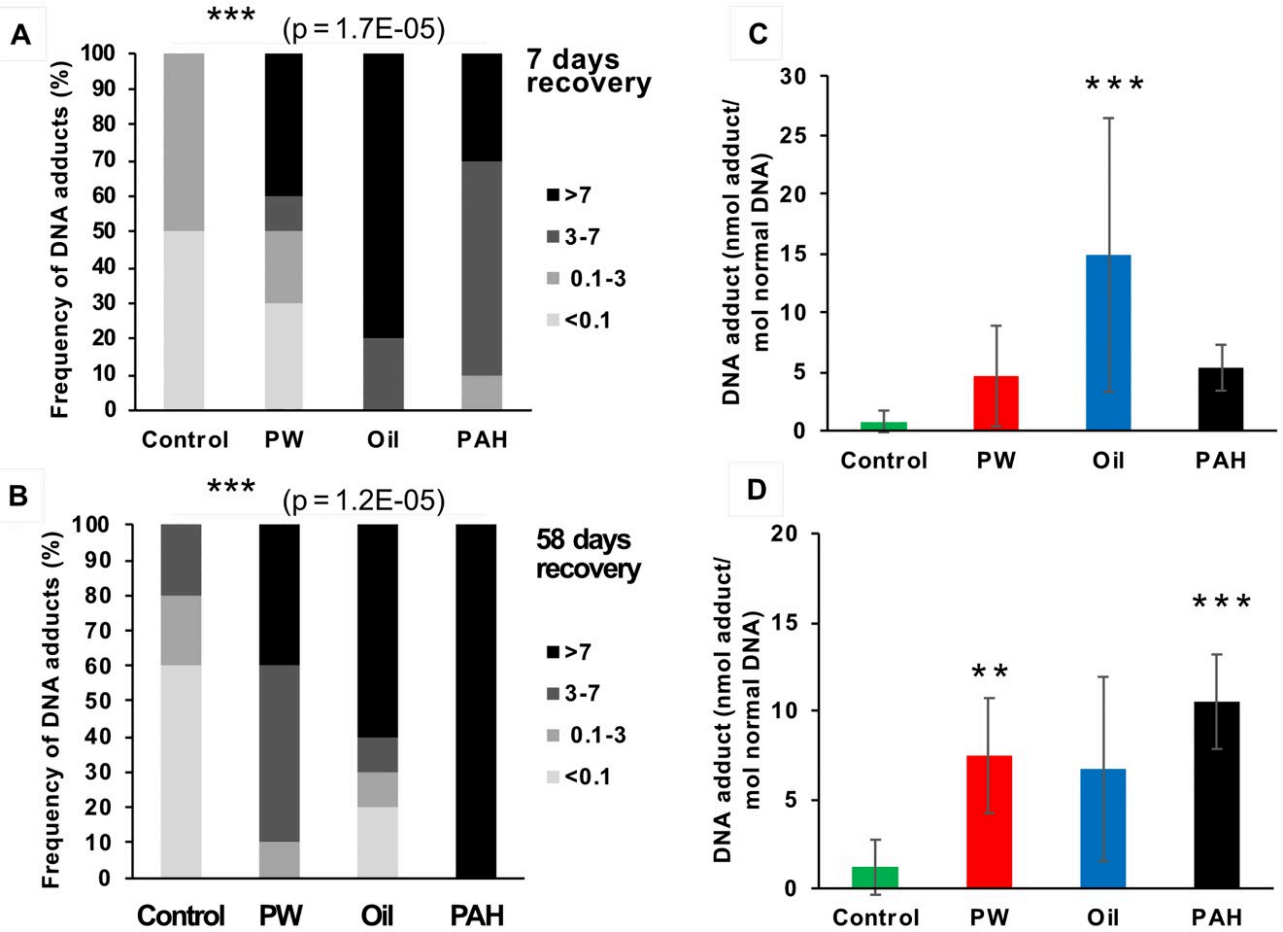
DNA adducts in the liver of fish from the different treatments groups after 7 day recovery (A and C, n = 10), 58 days recovery (B and D, n = 10). A-B are showing the frequency (%) of DNA-adduct divided into four intervals (below detection limit; <0.1 nmol DNA adduct/mol normal DNA, 0.1–3 nmol DNA adduct/mol normal DNA, 3–7 nmol DNA adduct/mol normal DNA, >7 nmol DNA adduct/mol normal DNA. Asterisks indicate statistical difference in the relative distribution between the treatment groups (Fisher Exact Contingency Table analysis). P<0.001 = ***. C-D are showing the average amount of DNA adduct (±SD). Asterisks indicate statistical difference to the control fish (Kruskal-Wallis Test). P<0.01 = **, P<0.001 = ***.

All three treatments (*PW*, *Oil*, and *PAH*) generally resulted in increased levels of DNA adducts in liver compared to the control, both in the exposure period (Fig 3) and in the recovery period (Fig 4), although the increases were not always significant. Sample results were divided into four intervals: below limits of detection (LOD) at <0.1 nmol DNA adduct/mol normal DNA; 0.1–3 nmol DNA adduct/mol normal DNA; 3–7 nmol DNA adduct/mol normal DNA; and >7 nmol DNA adduct/mol normal DNA. These intervals were chosen based on the recommendations from the International Council for Exploration of the Sea (ICES) Expert Group [35, 36].

DNA adducts levels in haddock liver varied substantially between treatments. In the control groups, 65% of the samples (N = 54) were below LOD of 0.1 nmol DNA adduct/mol normal DNA. Fewer samples were below LOD in the non-control groups: 31% in *PW*, 26% in *Oil*, and 2% in *PAH*. In the control group only one fish had DNA adduct levels above 7 nmol DNA adduct/mol normal DNA (approximate EAC) at any time point (2% of all control samples). Meanwhile, 22% of all *PW* samples, 37% of all *Oil* samples, and 63% of the *PAH* samples were above EAC.

At the first sampling point (after which the fish had only been given one day’s feed), all exposure groups had increased number of fish with detectable levels of DNA adducts in their liver. The *PAH* exposure, with the heavy 4/5/6-ring PAHs, resulted in a strong induction of DNA adducts (34±22 nmol adducts/mol normal DNA) compared with the control group (1±2 nmol adducts/mol normal DNA). This *PAH* treatment group also had elevated levels of DNA adducts compared to the control after 10 days. However, the level in *PAH* at day 10 (10±5 nmol adducts/mol normal DNA) was only about one-third the level that was observed in that treatment at day 3 (Fig 3B and 3F). During the exposure, the average amount of adducts in the fish, however, was significantly different from control only in the *PAH* treatment (Fig 3G and 3H).

No clear decline in the levels of DNA adducts during the recovery period was observed, and after 58 days’ recovery the three treatment groups still had higher DNA adducts levels (6.7–10.5 nmol adducts/mol normal DNA) than the control group (1.2 nmol adducts/mol normal DNA), although the higher levels were not always significant (Fig 4C and 4D). This shows that PAHs from all the different feed treatment groups are taken up orally, and that a single oral dose is sufficent to induce DNA adducts 3 days after exposure. This also shows that the clearance period may be very long before a group of exposed fish are back to a background level of liver DNA adducts. Initially, we observed disparate DNA adduct responses, but over time and through the recovery period, the treatments began to have more similar levels.

#### 3.3.3 Autoradiogram TLC maps

**Fig 5:**
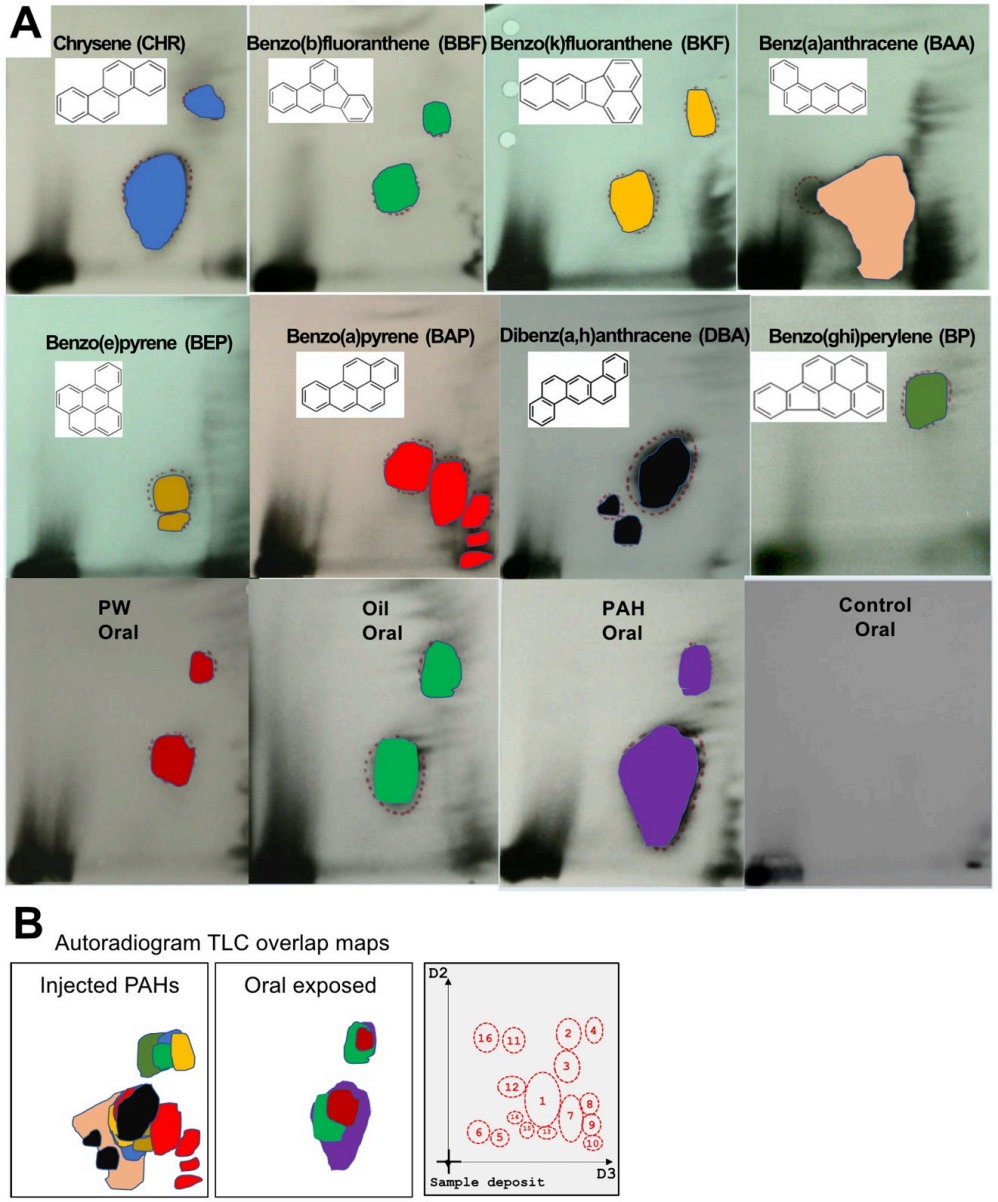
A. Representative autoradiogram of TLC maps in liver of haddock injected with single PAHs or exposed orally through feed for mixtures of PAHs (*PW*, *Oil*, *PAH*, Control). B. Overlap maps of the TLC spots from the different injected single compounds and the oral mixtures. 16 different spots from all the 384 plates (192 samples) analysed in this project were identified.

One main objective of this investigation was to investigate if different PAH sources yielded different DNA adduct patterns on the ^32^P-postlabelling TLC plates, and whether the spot position could be used to identify the sources of PAH exposure. Fig 5A shows the difference spot position of the heavy PAH single compounds and the three oral mixtures (duplicate radiographs are provided in S3 Fig in S1 File). When the spot profiles are overlain, we observed no unique TLC pattern that was indictive of an individual PAH or mixture (Fig 5B).

Among the fish dosed via injection, clear differences in the numbers of DNA adducts detected on the TLC plates were observed, with BAP giving rise to at least 5 different spots while BP treatment only shows one spot. There were some differences in the position of the spots from the different PAHs. However, the main picture is that there was a strong overlap between all compounds and no unique TLC pattern was revealed. Similarities in the pattern of spots between most PAHs were seen, with a big spot in the middle of the plate (spot 1) and several smaller spots in the upper right corner (spot 2, 3 and 4). No major spot with high intensity appeared to be specific to one single PAH compound. Spots 7, 8 and 9 were only observed in fish exposed to BAP. Spots 12, 14 and 15 were only observed in fish exposed to DBA. Spot 13 was only observed in the liver of one fish exposed to BEP (Fig 5B).

The TLC patterns of the oral exposed groups were less diverse compared to the single PAH injected groups. Three spots were found in more than 10% of all the samples, spot 1, spot 3 and spot 5. Table 4 shows a clear increased presence of DNA adducts in the exposed groups (*PAH* >*Oil* >*PW* >>control). However, it was not possible to distinguish between the three different PAH sources from the oral exposed groups using the TLC spot pattern.

**Table 4:** Frequencies of the total number of samples in each treatment groups with detectable levels of the three dominating spots (1, 3 and 5).

	Control group	*PW* group	*Oil* group	*PAH* group	Chi-square test
Spot1	21%	50%	66%	81%	P<0.0001
Spot3	0%	14%	19%	26%	P = 0.007
Spot5	17%	40%	48%	49%	P = 0.008

This confirms the limitation of the ^32^P-postlabelling assay to give structural information of the different DNA adducts, even when considering spot patterns. Thereby the identity of the PAHs that lead to DNA damage cannot be determined with this assay.

### 3.4 PAH bile metabolites

**Fig 6:**
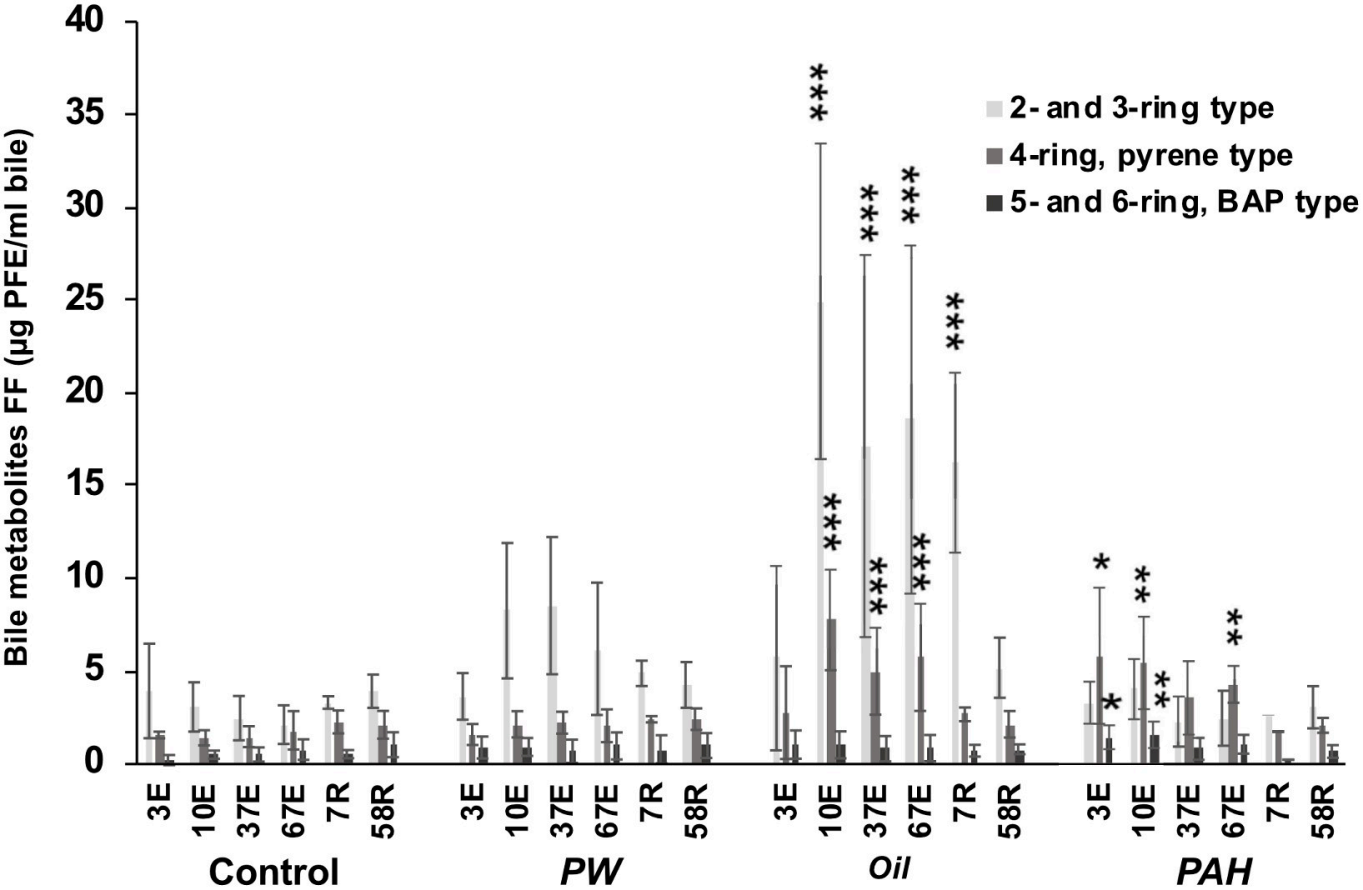
Fixed wavelength fluorescence (FF) analysis of PAH bile metabolites. FF were measured at the 290/334 nm (2/3 ring-type), P341/383 nm (pyrene-type) and 380/430 nm (benzo[*a*]pyrene-type). From the different treatments groups, control (C), produced water (PW), oil extracts (Oil) and heavy PAH mixture (PAH) after 3 day (3E), 10 days (10E), 37 days (37E) and 67 days (67E) of oral exposure, and after 7 days (7R) and 58 days (58R) recovery. Data presented as means (±SD). Asterisks indicate statistical difference relative to the control fish (ANOVA). P<0.05 = *, P<0.01 = **, P<0.001 = ***.

The measurement of PAH metabolites in bile is a well-established biomarker for exposure to oil and PAHs. The highest levels were observed in the *Oil* treatment, with strong induction of PAH metabolites of 2- and 3-ring type and 4-ring pyrene-type observed at several time points (Fig 6). In *Oil*, significantly higher levels versus the control group were seen as soon as 10 days of exposure, and remained high until the middle of the recovery period. After 7 days of recovery for *Oil*, only elevated levels of 2- and 3-ring type PAH metabolites were observed, but not of the 4 ring pyrene-type.

The *PAH* treatment group, with only 4/5/6-ring PAHs, had elevated amounts of both 4-ring pyrene type and 5- and 6-ring BAP type PAH metabolites already after 3 days and one single oral dose. After 67 days of exposure, only the 4-ring pyrene-type metabolite concentration was higher than control. Elevated levels of bile metabolites were not recorded in the recovery period. For the PW treatment group, we observed a trend, albeit insignificant, of higher average levels of the 2- and 3- ring type metabolites compared with control at 10, 37 and 67 days exposure.

### 3.5 PAH burden in liver

**Fig 7:**
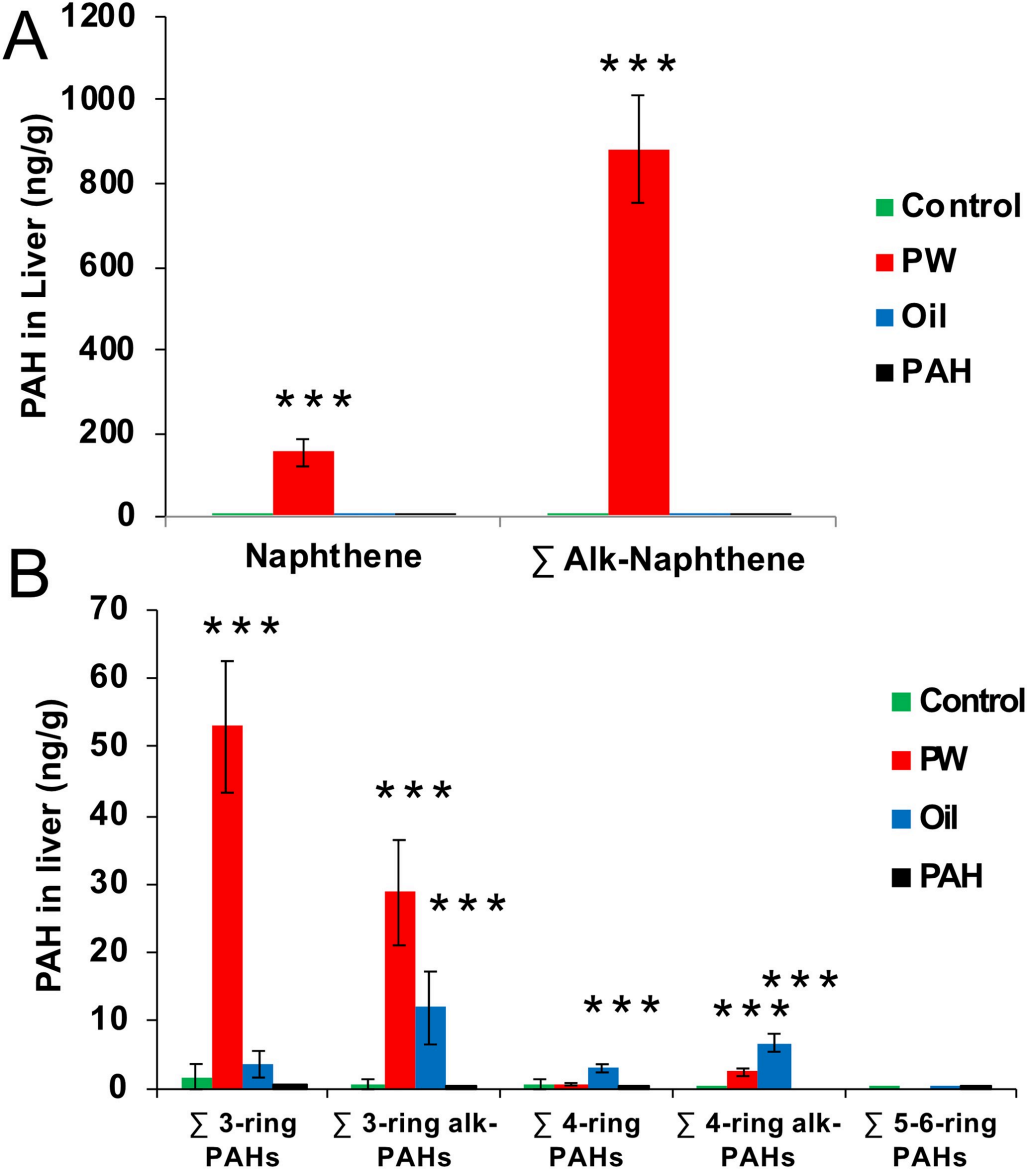
PAH found in the liver (ng/g) of the different treatments groups after 67 days of oral exposure. A. Naphthalene and sum alkylated naphthalenes. B. Sum 3-6-ring PAHs and their alkylated forms. Data presented as means (±SD). Asterisks indicate statistical difference relative to the control fish (ANOVA). P<0.001 = ***.

Burden of non-metabolised PAHs in the liver were analysed after 67 days of oral exposure to determine if PAHs were accumulating in the fat rich tissue. Results showed large amounts of PAHs in the liver of the *PW* treatment groups (Fig 7). The PAH profile of the livers of *PW* exposed fish were dominated by 2-ring, followed by 3-ring PAHs, and with a small but significant amount of alkylated 4-ring PAHs. Three-ring alkylated PAHs and 4-ring PAHs (both alkylated and non-alkylated) were also found in the *Oil* treatment, though in much lower amounts. No heavy PAHs were detected in the *PAH* treatment group.

As a comparison, a theoretical calculation was done regarding how much the total liver content of PAHs (concentration (ng/g) x liver size (g)) was related to a single dose with food (1% of body weight). In the *PW* group, the naphthalene content in the liver corresponded 34% of the total PAHs in one dose, for the alkylated 2-ring PAHs it corresponded to 16%, for the 3-ring PAHs it was 51% and for the alkylated 3-ring PAHs it was 12%. The similar calculation for the *Oil* group showed that a much lower part of the total PAH dose was found in the liver; 3-ring PAHs were 2.4%, alkylated 3-ring PAHs were 0.1% and for the 4-ring PAHs it was 0.5%. For both the *PW* and the *Oil* group lower amounts of alkylated PAHs were found compared with the non-alkylated PAHs, suggesting a faster clearance rate for alkylated PAHs.

### 3.6 Effects on selected biomarkers

Different biological effects at the cellular level relevant for PAH exposures were measured in the liver of exposed fish and the controls, including qPCR (to measure mRNA levels of *cyp1a*, *ahrr*, *GADD45A*, *GADD45G*, *P53*), ELISA to measure protein levels (CYP1A), and measurements of GST activity and lipid peroxidation in membranes. Treatment-induced effects were observed with *cyp1a* and *ahrr* expression and CYP1A protein levels, however the other biomarkers had no significant treatment-related effects. Effects measured in the intestine are described in Section 3.7.

**Fig 8:**
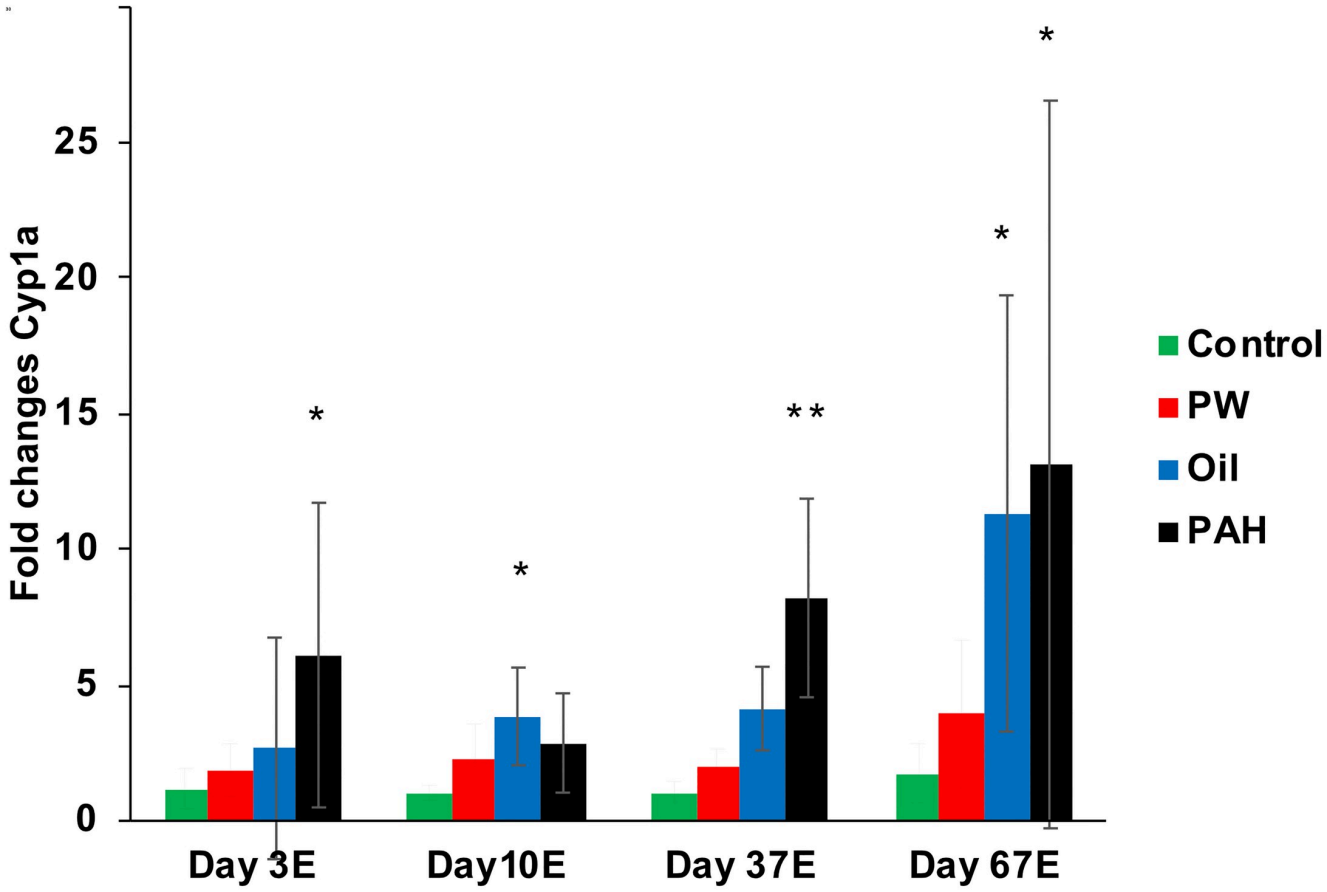
Gene expression of *cyp1a* in liver after 3 day (one dose), 10 days, 37 days and 67 days of oral exposure. Data presented as means (±SD). (*) indicate significant differences compared with control, p< 0.05.

Expression of *cyp1a* was strongest in the *PAH* exposure group, and it was significantly induced compared with the control after 3 days (one dose), 37 days and 67 days of exposure (Fig 8). The *Oil* exposure group also showed significantly induced levels of *cyp1a* gene expression after 10 and 67 days of exposure. No significant induction was observed in the *PW* group. Following these trends, ELISA analyses of CYP1A protein levels in liver (after 67 days of exposure) showed the same pattern with the cyp1a gene expression data from qPCR analyses, with significantly increased levels of CYP1A protein in the PAH and oil treatment group. Although, the relative differences from control to exposed groups were higher in the qPCR analyses than in ELISA (S5A Fig in S1 File), due to background staining in the control group when using ELISA. Additionally, the aryl hydrocarbon receptor repressor, *ahrr*, was significantly induced in the PAH and oil treatment groups after 67 days of exposure (S4 Fig in S1 File).

There were no observed effects in the remaining biomarkers. DNA repair systems were assessed using several primers to transcripts, including growth arrest and DNA damage inducible proteins (GADD) involved in the P53 signaling pathway (*gadd45a*, *gadd45*G and *p53*). No significant difference in transcript response of cellular DNA repair systems was observed in any group compared to the control after 67 days of exposure (S4 Fig in S1 File). There was also no significant effect on *gst* from the exposures, which was included to investigate effects on the phase-2 detoxification proteins (S5B Fig in S1 File). Finally, measurements of lipid peroxidation as an indicator of oxidative stress did not reveal significant changes in the exposed groups compared with control (S5C Fig in S1 File).

### 3.7 Effects in the intestines of orally-exposed haddock

#### 3.7.1 DNA adducts in intestine

**Fig 9:**
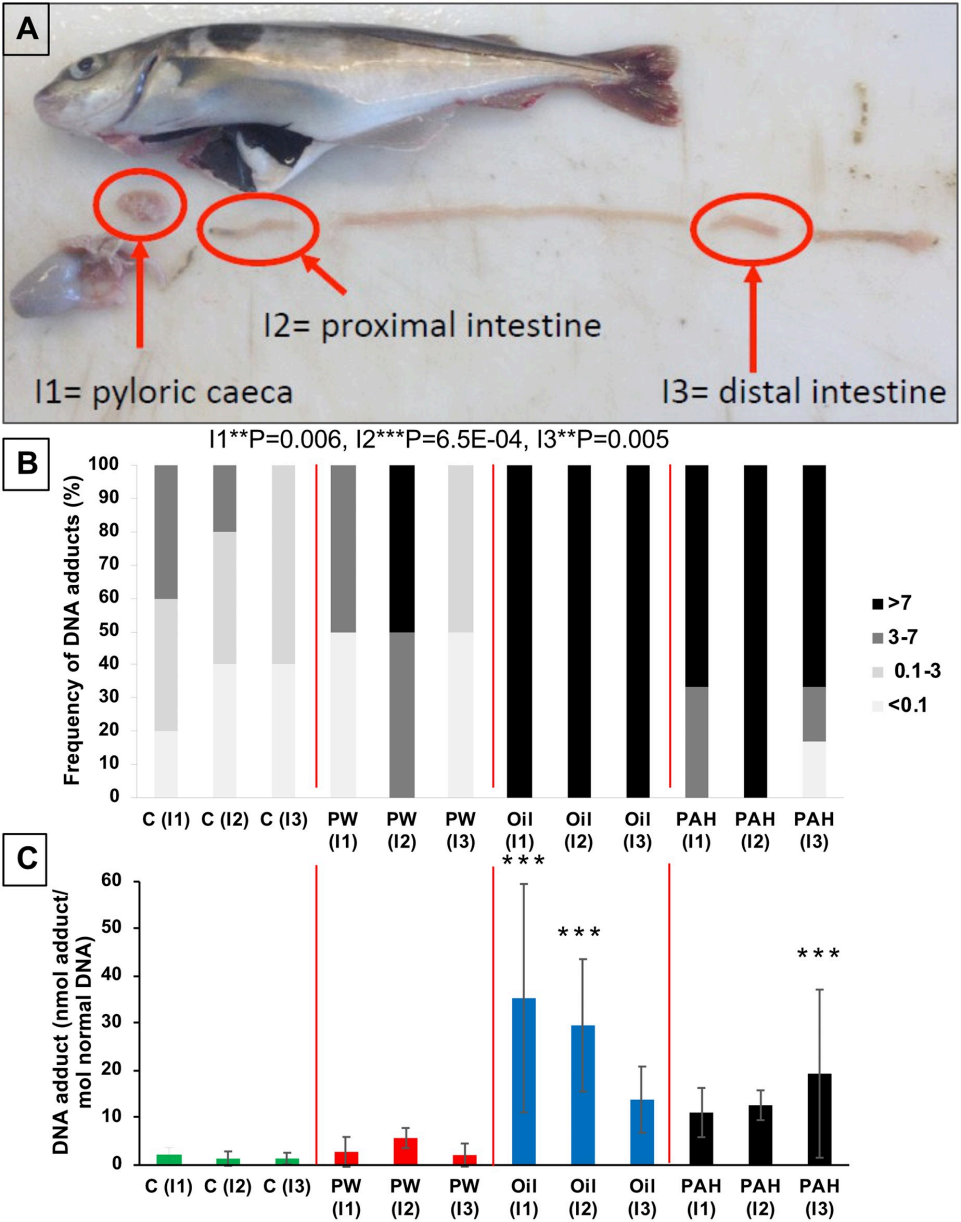
DNA adducts in the intestine system of fish from the different treatments groups after 67 day of oral exposure to three exposure feeds; produced water (*PW*, n = 4)), oil fraction (*Oil*, n = 4) and heavy PAH mixture (*PAH*, n = 6) and a clean control feed (C, n = 5). Samples from different parts of the intestine were taken at 3 different positions (A). (B) The frequency (%) of DNA-adducts divided into four intervals (below detection limit; <0.1 nmol DNA adduct/mol normal DNA, 0.1–3 nmol DNA adduct/mol normal DNA, 3–7 nmol DNA adduct/mol normal DNA, >7 nmol DNA adduct/mol normal DNA. Asterisks indicate statistical difference in the relative distribution between the treatment groups (Fisher Exact Contingency Table analysis). P<0.01 = **, P<0.001 = ***. (C) the average levels of DNA adducts (±SD) (number of samples are given in brackets). Asterisks indicate statistical difference to the control fish (Kruskal-Wallis Test). P<0.001 = ***.

The formation of DNA-adducts in three different parts of the intestine was measured after 67 days of oral exposure: the pyloric caeca, the proximal intestine, and the distal intestine (Fig 9A). Frequencies of strong induction of DNA adducts were significantly higher in the *Oil* and *PAH* treatment groups compared to the control from all the parts of the intestine system (Fig 9B). All samples from all three parts of the intestine from the *Oil* treatment had adduct levels above 7 nmol DNA adduct/mol normal DNA, the approximate EAC. For the *PW* treatment groups, neither the levels of DNA adducts nor the frequency of induced fish were different the control. The *Oil* treatment group had significantly higher DNA adduct levels in the first part of the intestine (pyloric caeca and proximal intestine), but not in the distal intestine (Fig 9C). Conversely, the *PAH* treatment had significant higher levels of DNA adducts in the distal intestine, but not in the front of the intestine. In the *PW* treatment group, no significantly increased DNA adducts levels were observed compared with the control groups in any of the intestine samples (Fig 9C).

#### 3.7.2 *Cyp1a* induction in intestine

**Fig 10:**
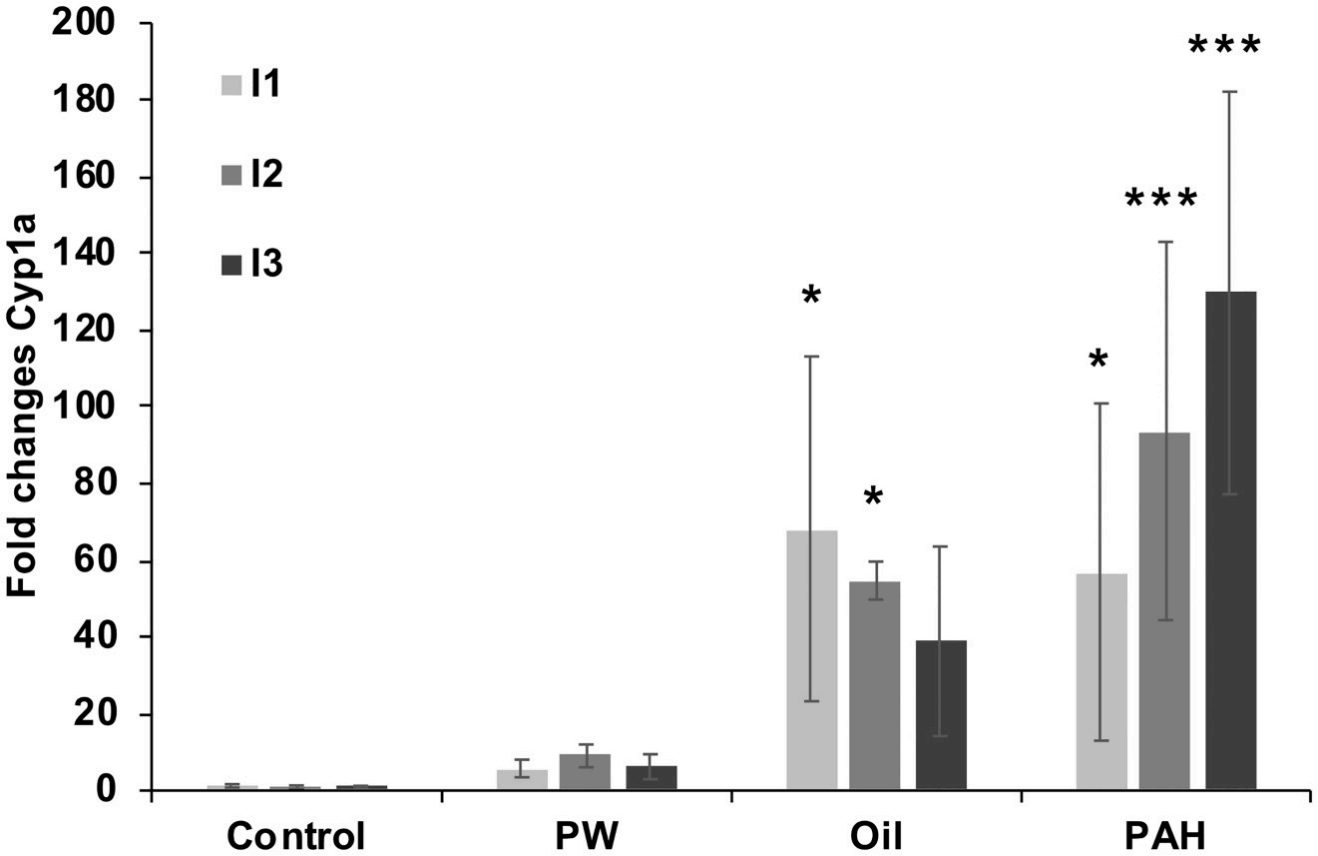
Gene expression of *cyp1a* in the intestine system of fish from the different treatments groups after 67 days of oral exposure to three exposure feeds; produced water (*PW*, n = 4), oil fraction (*Oil*, n = 4) and heavy PAH mixture (*PAH*, n = 6) and a clean control feed (C, n = 5). Samples from the intestine system were sampled at 3 different positions (I1 = pyloric caeca, I2 = proximal intestine, I3 = distal intestine). Data presented as means (±SD). Asterisks indicate statistical difference relative to the control fish (ANOVA). P<0.05 = *, P<0.001 = ***.

In the *Oil* treatment group, a highly increased gene expression of *cyp1a* was found in the first part of the intestine (pyloric caeca, I1 and proximal intestine, I2), but not in the distal intestine, I3 (Fig 10). For the *PAH* treatment group, all positions in the intestine had significantly increased *cyp1a* expression. For the *PW* treatment group, there was no increase in the *cyp1a* expression in the intestine.

### 3.8 Histopathology and macroscopic liver lesions

At the end of the exposures, fish with grossly visible or macroscopic liver lesions (potentially necrosis, tumors/nodules, areas of swelling and fluid-filled cysts) were observed, mainly in the *PAH* exposed groups, but also in *PW* and *Oil* (Table 5, S6 Fig in S1 File). One should be very careful in drawing conclusions from this kind of macroscopic observation; however, these gross observations suggested that PAH treatments induced damages to liver physiology and tissue structure, so a subsequent histopathological evaluation was therefore prioritized for further studies. After 67 days of exposure, 10% of individuals (2 out of 20 fish) in the PAH exposed group contained large areas of what grossly appeared to be necrotic tissue, and after one week of subsequent recovery the possible necrotic tissue was also seen in the *PW* group (2 out of 10 fish). In the PAH exposed group, livers in 2 of 10 fish contained large fluid-filled cysts, and one fish grossly exhibited a large mass or nodule/tumor, which could not be confirmed as a neoplasm by histopathological examination. Because of the low number of fish that were sampled, after 67 days of exposure and subsequently at 7 days of recovery from exposure, it is difficult to predict if the frequencies of liver damage were truly representative. After a two-month recovery on clean food, 31–35 fish were sampled, and only a few fish (≈3%) had clearly visible macroscopic liver lesions.

**Table 5:** Numbers of fish observed with macroscopically visible liver lesions after 67 days of exposure (67E), and after 7 (7R) and 58 days (58R) of recovery.

Treatment	Day 67E	Day 7R	Day 58R
Control	0 of 20	0 of 10	0 of 36
*PW*	0 of 20	2 of 10	1 of 35
*Oil*	0 of 20	0 of 10	2 of 31
*PAH*	2 of 20	3 of 10	1 of 33

No statistical differences from control were observed (Fisher’s exact test).

Samples were taken randomly from the liver and fixed for histopathological examination with the goal of detecting various potentially neoplastic, preneoplastic or other distinctly non-neoplastic lesions. Histopathological studies can be difficult and there is always a risk of misdiagnoses [37]; therefore a double evaluation was performed here as recommended. Samples were examined blindly at NORCE, and those results were further evaluated by a detailed, blind histopathologic examination by Mark Myers. At the first evaluation, a high prevalence of necrosis was recorded in all samples (>50% in both control and exposed groups). However, the detailed re-examination and analysis revealed that the majority of these previous observations represented artefacts of post-mortem autolysis and necropsy-related tissue trauma.

The histopathological examination did not reveal any significant increase in malformations (Table 6). We observed a tendency of increased, but insignificant, necrosis and inflammation in the PAH treated fish at the end of the two-month exposure and also after one week of recovery. No neoplasms (tumors) or preneoplastic hepatic lesions (e.g. eosinophilic focus) were observed in fish examined from any of the exposed or control groups. These results suggest that haddock may have a high capacity to repair previously caused liver lesions and damage.

**Table 6:** Morphologic diagnostic results from histopathological examination of liver in haddock after 67 days of exposure, 7 days of recovery and 58 days of recovery.

	Macrophage aggregates	Granulomas	Circulatory distrubances	Vacuolated areas	Steatosis/fatty change	Fibrosis/cirrhosis	Necrosis	Eosinophilic focus	Mononuclear inflammatory cell infiltrates
**67 days exposure**									
C	100	67	11	0	0	0	0	0	0
*PW*	100	56	0	0	0	0	0	0	0
*Oil*	89	56	22	0	0	0	0	0	0
*PAH*	100	67	2	0	0	11	22	0	11
**7 days recovery**									
C	100	56	33	0	0	0	0	0	11
*PW*	100	56	0	0	0	22	22	0	0
*Oil*	100	67	0	0	0	0	0	0	0
*PAH*	100	67	0	0	11	44	33	0	33
**58 days recovery**									
C	100	78	11	0	0	0	0	0	0
*PW*	100	67	11	0	0	0	0	0	11
*Oil*	100	56	11	0	0	0	0	0	0
*PAH*	100	67	22	0	0	0	0	0	11

Nine fish were examined from each group each time, the results show the percentage of fish that were positive for the different measurements. No statistical differences from control were observed (Fisher’s exact test).

### 3.9 Vertebral malformation in X-rays

**Fig 11:**
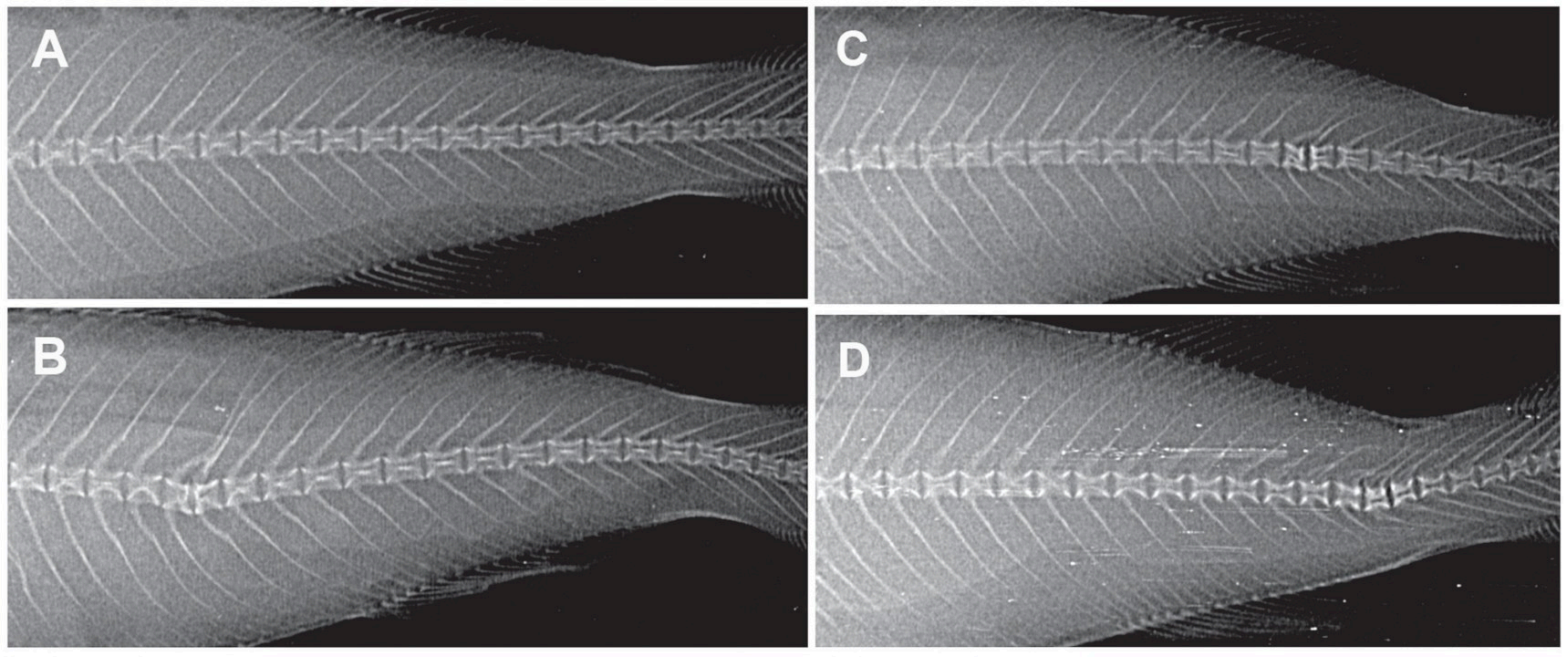
X-ray pictures of haddock from Control (A), *PAH* (B) and *Oil* (C and D). The picture shows different examples of vertebral malformations; deformities in vertebral 25–27 (B), 34–36 (C) and 36–39 (D).

The treatments had a significant effect on the occurrence of fish with deformed vertebrae (Chi-square test, p-value <0.01, Table 7). Twenty fish per treatment were radiographed at one time point, at the end of the 67 days of exposure (Fig 11). These preliminary results show that this vertebral malformation may be a result of exposure to compounds in oil.

**Table 7:** Frequency of malformation in PAH exposed haddock.

	Number of fish with vertebra deformities	Deformity rate (%)
Control	1	5
*PW*	4	20
*Oil*	6	30
*PAH*	6	30

From each group 20 fish were examined.

## 4. Discussion

There are at least two main categories of PAHs that fish can be exposed to in open sea; small 2-4-ring PAHs with a petrogenic origin and larger 4/5/6-ring PAHs of pyrolytic origin. The route of exposure may be uptake directly from the water, uptake from contact with (or ingestion of) contaminated sediments or orally through the food chain. All three exposure routes may be relevant for haddock which is a demersal fish, finding its food in or just above the sediments. In this study, we have focused on oral exposure, comparing three different PAH mixtures with the intention to represent three different contamination sources; 2-ring PAHs extracted from *PW*; 3- and 4- ring PAHs obtained from crude *Oil* distillation fractions and representing oil contaminated drill cuttings; and heavy 4/5/6-ring *PAH*s representing background in sediments contaminated from atmospheric fallout and urban runoff. We also injected fish with heavy PAHs to understand patterns of DNA adducts induced from distinct PAHs and mixtures.

### 4.1 DNA adducts and health effects

#### 4.1.1 DNA adducts

The formation of DNA adducts found in haddock after intraperitoneal exposure to selected PAHs agrees well with how the PAHs are characterized by the International Agency for Research on Cancer (S3 Table in S1 File) [14]. The four PAHs that lead to high levels of DNA adducts in haddock liver, BAA, BAP, CHR and DBA, are all known to be potent inducers of DNA adducts and also to be carcinogenic or possible carcinogenic compounds [38, 39]. Meanwhile, FL, PY and IND did not induce DNA adducts and are classified as non-carcinogenic [38, 40]. Five PAHs had detectable but low levels of DNA adducts (BEP, BBF, BKF, PER, BP). BBF and BKF are suspected to be carcinogenic, but none of them are known to be very potent to induce DNA adducts [38, 40–42].

Oral exposure for complex PAH mixtures from *PW* extracts (2-ring PAHs), *Oil* extracts (3-ring PAHs) and heavy *PAH* (12 compounds of 4/5/6-ring PAHs) showed that all PAH mixtures, both the petrogenic and the pyrolytic type, induce DNA adducts in haddock when given through diet. The levels of DNA adducts in the present study were similar to those found in wild haddock [28]. In the North Sea, it is expected that demersal fish experience water-borne exposures in addition to oral intake while bottom-feeding. The smaller 2- and 3-ring PAHs like those in *PW* treatment are more soluble in water, while the heavier PAHs in *Oil* and *PAH* are more likely to be adsorbed to organic material and taken in as food [43]. From our studies, we saw levels of DNA adducts that are comparable with what has been observed for wild fish, despite only using oral doses in the experimental design.

Similar studies have been performed with water exposure of oil and PW. Aas et al. [18] exposed Atlantic cod (*Gadus morhua*) to crude oil through the water (0.06–1 mg oil/l = 0.33–7.8 μg PAH/l) and found an induction of liver DNA adducts (11±4 nmol adducts/mol normal DNA) in the high exposure group after 3 days of exposure. DNA adduct levels increased during the 30 day experiment, peaking at 109±45 nmol adducts/mol normal DNA at the end of exposure. Levels of DNA adducts did not decline during a seven day recovery period in clean water. The low doses of oil (0.33μg PAH/l) also resulted in elevated DNA adduct levels (4±2 nmol adduct/mol normal DNA) after 30 days exposure [18]. In a similar 14 day water exposure study with crude oil (1 mg oil/l) on Atlantic cod and polar cod (*Boreogadus saida*), the same research group did only find medium induction of DNA adducts 18±11 and 12±4 nmol adduct/mol normal DNA respectely [44]. Long-term water exposure (16–44 weeks) of Atlantic cod to artificial PW (5.4 μg PAH/l + 11.4 μg alkylphenols/l) showed medium DNA adduct levels (9 nmol adduct/mol normal DNA) after 16 weeks of exposure, but high induction of DNA adducts after 44 weeks (73 nmol adduct/mol normal DNA) [19]. Atlantic cod exposure for PW for 28 days had low but significant induction of DNA adducts in 0.5% PW (4.4±2.4 nmol adduct/mol normal DNA) [21].

DNA adduct levels observed in PAH exposed haddock in the present study were in the same range as what have been reported in wild haddock captured around oil installation in the North Sea [7, 11]. Pampanin et al. [28] have reviewed the results of the Norwegian Offshore Condition Monitoring program 2002–2011, and in haddock collected around the large oil fields in the North Sea there are reported liver DNA adduct from <0.1–37 nmol adduct/mol normal DNA. In reference areas with no oil exploration, background DNA adduct levels in haddock liver are <0.3–1.5 nmol adduct/mol normal DNA (pristine area around Island), <0.3–4.8 nmol adduct/mol normal DNA (the Barents Sea) and <0.1–12 nmol adduct/mol normal DNA (Egersund bank, North Sea).

DNA adducts are considered a crucial biomarker of exposure in human and sentinel organisms, especially for their early emergence after a genotoxic exposure, which may play a key role in establishing a mode of action for cancer [45]. A strong correlation with detection of hepatic DNA adducts and increased frequency of severe toxicopathic liver lesions, including neoplasms, has been demonstrated in flatfish caught in heavily PAH contaminated areas reported by [10, 46, 47]. DNA adducts in fish can be very persistent and be detected several months after fish have been moved into a clean environment either from laboratory exposure [41, 48] or from PAH hotspots in the field [49], as also shown in this study. The mechanism behind the long turnover time for bulky DNA adduct in fish is not known. Fish have like most other organism a well-developed nucleotide excision repair (NER) machinery and should be able to repair DNA damage [50]. One explanation could be that the long-lived DNA adduct are located in the none-coding parts of the genome that are not so accessible to repair [51].

ICES assessed available documentation on DNA adducts in eight important marine fish from the North Atlantic and determined acceptable background concentrations for haddock to be 3 nmol DNA adduct/mol normal DNA). ICES also determined EAC, which is the concentration below which unacceptable biological effects are unlikely to occur [35, 52]. The EAC for haddock was determined to be 6.7 nmol DNA adduct/mol normal DNA [36, 52]. These ISES-determined levels were the basis for our interpretation of our DNA adduct results.

In the present study, we report clear evidence that the treatments resulted in an increased number of fish with liver DNA adducts above EAC (control = 2%, *PW* = 22%, *Oil* = 37% and the *PAH* = 63%, of 54 analysed fish from each treatment group). Exposures were confirmed with measurements of PAH and PAH metabolite burden in liver. However, we did not observe an increased mortality, neither acute during exposure, nor delayed after 58 days of recovery period. We observed some effects that can be related to fitness, since the two fish groups that were exposed for oil and heavy PAHs grew less than the control fish and the PW exposed fish, and were significantly smaller at the end of the recovery period. However, linear regression of DNA adducts versus daily growth factor for pooled samples from the one and two month exposure and the recovery period showed no correlation (*Oil*, R^2^ = 0.0009; *PAH*, R^2^ = 0.043); not all samples were analysed for DNA adducts, therefore only 40 samples out of 80 fish could be included in this analysis.

Reduced growth after oral exposure of PAHs and crude oil is well-documented and reported for several fish species. It has been observed in zebrafish (*Danio rerio*) after exposure to sediment extracts [53]; in rockfish (*Sebastes schlegeli*) exposed for BAP for 30 days [54]; in juvenile turbot (*Scophthalmus maximus*) following oral exposure for water-accommodated-fraction (WAF) of heavy oil [55]. Reduced growth has also been reported in juvenile Chinook salmon (*Oncorhynchus tshawytscha*) fed pellets mimicking PAH exposure from urban estuaries [56]; in polar cod given oil contaminated feed [57]; and finally, in polar cod force-fed crude oil [58]. Reduced growth can lead to lower fecundity and increased risk of being predated [59, 60].

#### 4.1.2 Responses on selected biomarkers

AhR repressor (AhRR), inhibits AhR function by competing with AhR for dimerizing with Arnt and binding to the XRE sequence. Thus, AhR and AhRR form a regulatory circuit in the xenobiotic signal transduction pathway and provide a regulation of AhR function [61].

As strong effects on DNA adducts were observed, we included primers for gene transcripts implicated in genotoxic stress-induced responses like growth arrest and DNA inducible 45a (Gadd45a), gadd45g and P53 [62, 63]. However, we did not observe significant changes in expression levels between the different treatments.

Lack of responses in GST levels or LPO is probably due to lower responsiveness in these biomarkers compared with CYP1A and AHRR. Results with GST expression on larvae of Atlantic cod indicated GST expression to be only moderately inducible [64]. Also, GST and LPO levels were not significantly different in juvenile cod or golden grey mullet exposed to oil [65, 66].

#### 4.1.3 Histological changes

Macroscopic liver damage and histopathologic changes were observed at end of the exposure and after the recovery period. However, by the end of the recovery period at 58 days, we no longer observed necrosis in any group and only few differences in the liver cell physiology were found when comparing the different treatment groups. These results indicate that the long-term PAH exposures induce pathological effects in the liver, but that that haddock do have the capacity to repair such liver damages.

Macro-evaluation of fish liver during sampling revealed presence of abnormalities in individuals in the *PAH* group (e.g. large water cysts, necrosis, blood infiltrations). None of these abnormalities was noted in fish from the control group. Due to the low number of fish that were sampled after 67 days (20 fish per treatment) and after 7 days of recovery (10 fish per treatment), it is difficult to predict whether the frequencies of liver damages were representative. After two months of recovery, 31–36 fish were sampled per treatment, and only a few fish (≈3%) were observed with clear visible liver damages. Histopathological examinations confirmed the macroscopic observation in the *PAH* treated fish both at the end of the two months of exposure and after 7 days of recovery.

#### 4.1.4 Bone mineralization

It is well known that oil and PAH exposure on early life stages severely affects the bone formation [67–70]. Using fish scales as a model, PAHs and their hydroxylated metabolites are shown to disrupt bone metabolism in fish [71–73]. Vertebra bone mineralization has therefore been suggested as a biomarker of PAH pollution in adult marine fish [74]. Bone deformities in wild fish have for decades been used to monitor aquatic pollution [75–77]. Vertebral column deformity has been found in adult wild haddock caught in a Norwegian fjord [78], but no clear pollutant source was identified. In this study, we found an increased number of haddock with vertebra deformities from all three exposed groups compared to controls. Even though our observations only were done at one time point (67 days of exposure) with a limited amount of fish (n = 20 in each treatment groups), we will recommend to include radiological deformity screening to be considered for future environmental monitoring of O&G related activities.

### 4.2 The 32P-postlabelling assay is not able to distinguish between different PAH exposures

The ^32^P-postlabelling assay is the preferred analytical method for studying DNA adducts because of its very high sensitivity [27]. However, this method has limited capacity to identify which PAHs (or other compounds) are responsible for the formation of DNA adducts. Knowing this limitation, we attempted to extend the utility of the assay by evaluating and comparing patterns among exposures to single PAHs and mixtures. However, we were not able to reliably differentiate the exposure treatments, or the identity of DNA adducts, based on the results of the assay.

As discussed in Pampanin et al. [28], it is mandatory to develop specific and sensitive MS methods for oil and PW related DNA adducts, to be able to identified the sources of pollution in the North Sea that are responsible for the formation of DNA adduct in wild fish populations. During the last decade, several research groups have aimed to develop MS methods for analysis of DNA adducts [79, 80]. However, an important challenge has been to get analytical platforms that are able to achieve the extremely low detection limits needed (1 adduct among 10^9^ unmodified DNA bases). New developments in LC-MS technology have made possible the necessary demands for sensitivity, and a significant effort has been put into the development of new MS based methods for identification of DNA adducts [81–84]. Samples from the present study are now being used for a method development; first for identification of which PAHs (or other compounds) that are bound to the DNA in the DNA adduct; and later when standards have been synthesised, to make quantitative methods to change the offshore monitoring from using the ^32^P-postlabelling assay to a LC-MS assay.

### 4.3 Uptake and metabolism of PAHs through oral exposure reveal the importance of intestinal detoxification

Bile analyses of PAH metabolites were performed using the FF screening method, which is a well-established biomarker for oil and PAH exposure [85]. As expected, high levels of 2- and 3-ring PAHs and pyrene-like PAHs (4-ring) metabolites were found in the bile from the *Oil* exposed fish. This was observed from day 10 and remained high during the whole exposure period through 7 days of recovery. However, it returned to background levels after 58 days of recovery. This fits well with what is normally observed with oil exposure in fish [18, 30, 86]. In the *PAH* treatment groups, we found significantly elevated levels of pyrene-like and BAP-like (5- and 6-ring) metabolites in the bile already after 3 days (and only one dose) of exposure, and also after day 10. However, in the later sampling points, only pyrene-like metabolites were found to be significantly elevated at the end of exposure. This is in accordance with observations in juvenile rainbow trout (*Oncorhynchus mykiss*) fed pellets containing a mixture of heavy PAHs [87].

Metabolism of PAHs is catalyzed via the cytochrome P450 monooxygenase system. It is commonly found that oil exposure in fish results in a strong induction of Cyp1a which in turn increases the metabolism of PAHs [18]. Many heavy PAHs, (4–6 rings) are very strong CYP inducers; likewise alkylated 3-ring PAHs have also been shown to bind to AhR and induce the CYP systems [88]. The small 2-ring PAHs, like those predominating the *PW* exposure, are *not* agonists for AhR, which would be responsible for the induction of the metabolic enzymes (cytochrome P450, CYP) for PAHs. Despite the 2-ring PAHs dominating in PW, significant Cyp1a induction has nevertheless been observed in PW exposed cod [21, 86], although at much lower levels compared with crude oil exposure. In the present work, we did not find significantly elevated levels of PAH metabolites in *PW* exposed at any of the sampling points. And of the two treatments that were dominated by heavier PAHs and induced CYP systems, we did find significant increases in metabolites.

GC-MS/MS measurements showed high levels of non-metabolised naphthalenes (2-ring) and phenanthrenes (3-ring) in the liver of the *PW* exposed fish. Small amounts of 3- and 4-ring PAHs were found in the *Oil* exposed fish, while no heavy PAHs were detected in the *PAH* treated groups. These results also align well with the hepatic Cyp1a system, which was only strongly induced in the *Oil* and the *PAH* treatment groups, but not in *PW*. Although, the presence of *such* high levels of 2- and 3-ring PAHs in the livers *PW* exposed fish was surprising because non-metabolised PAHs are normally only found in low levels in fish tissues like liver and muscle [89].

Detoxification via AhR pathway and CYP mechanisms is strongly inducible in fish, but what target tissue that will be affected is highly determined by the route of exposure. The liver is the most studied organ and has high capacity of metabolising PAHs. Through intraperitoneal injection or water exposure of PAH, it is normal to find a strong induction of Cyp1a in the liver of fish. However, for oral exposures, we expected to find the strongest induction of Cyp1a in intestine. Several studies have found that the amount of BAP (5-ring PAH) that reaches the liver is much lower in oral exposure compared with exposure through intraperitoneal injection because the intestine accumulates and metabolises BAP, as shown for sea bass (*Dicentrarchus labrax*) [90], English sole (*Parophrys vetulus*), starry flounder (*Platichthys stellatus*) [91] and rainbow trout [92]. Comparisons between oral and waterborne exposure of BAP in mummichog (*Fundulus heteroclitus*) [93] and Nile tilapia (*Oreochromis niloticus*) [94] have shown that the induction of CYP1A detoxification are distributed quite differently within the organism after the two different exposure routes. In the orally-exposed fish, increased *cyp1a* gene expression and enzymatic activity were found only in the intestine, while such induction was only in the gill and liver of the water-exposed fish. Differences in body distribution between oral exposure to BaP and phenanthrene, has also been reported in Atlantic salmon *(Salmo salar)* [95] and polar cod [96], using a single oral dose of radioactive marked ^14^C-BAP and ^14^C-phenanthrene. BAP was mainly seen in the intestine, while phenanthrene was found in higher concentration in the liver and the bile. High accumulation of non-extractable BAP, i.e. covalently bound, like DNA adducts, were found to be accumulated in the intestine of both fish.

The first metabolism through the intestinal barrier is found to be very important for the protection against heavy PAHs like BAP (reviewed for fish in Van Veld [97] and mammals in Ramesh et al. [98]. In rodents, Cyp1a induction in the small intestine is crucial for protection against oral exposure of carcinogenic PAHs [99–102]. Human intestinal cells have similarly been found to have high capacity to metabolise heavy PAHs and transport them to be eliminated through the intestinal lumen. Thereby, PAHs are not taken up by the blood and will not reach the liver [103, 104]. The induction of the intestinal detoxification system is an important protection against oral exposure of these and similar compounds [99–102].

Results from the present study showed higher levels of DNA adducts in the intestine compared with liver for the *Oil* and *PAH* groups. No increased levels of DNA adducts (in neither liver nor intestine) were found initially in the *PW* group during exposure period, although increased adduct levels in liver were detected at the end of the recovery period. The Cyp1a was strongly induced in the intestine of the *Oil* and *PAH* groups, but not in the *PW* group. A clear difference between the *Oil* and the *PAH* group was observed; *Oil* (mostly 3-ring PAHs) showed the highest induction of DNA adducts and Cyp1a in the first part of the intestinal system (the pyloric caeca and the proximal intestine), while *PAH* (4/5/6-ring PAHs) showed the highest DNA adduct and Cyp1a induction in the distal intestine.

We collected intestine samples only at the end of the exposure, so we cannot remark on the induction of the detoxification system and the DNA adduct formation over time in the intestine. However, it seems likely that PAH metabolism in the intestine has played an important role for the results we observed in this study. It might explain why we found highest DNA adduct levels in liver from heavy *PAH*s exposed fish at day 3 after only one dose. One explanation might be that the detoxification system in the intestine was not fully induced (because exposures had only just begun) and therefore a larger part for the PAHs were taken up and transported to the liver. This is supported by findings of high induction of liver Cyp1a and the largest amount of bile metabolites (both 4 and 5-ring PAHs) in this group after 3 days. PAH metabolites were still detectable in the bile at day 10, but DNA adduct levels in the liver had declined to only 1/10 of what were found at day 3. Significantly increased levels of 5 ring PAH metabolites were not observed in the PAH groups after day 10, although the levels of 4 ring metabolites were still found to be increased compared with the control fish after the full 2 months of exposure. This might be explained by induction of Cyp1a in the intestine and that only a lower fraction of PAHs are transported to the liver, and that the majority of PAHs are metabolised in the intestine and excreted directly into the intestine.

Like the *PAH* treatment, the *Oil* group also had high induction of PAH metabolites and high levels of DNA adducts in the intestine. However, in contrast to the heavy *PAH* exposure, a large amount of PAH bile metabolites were seen in the *Oil* group all the way through exposure and also after 7 days of recovery with non-contaminated food. This study shows that there are differences in the metabolic pathways for heavy PAHs (≥5 rings), and small PAHs (2-ring and 3- to 4-ring PAHs) after oral exposure. Exposure for only 2-ring PAHs in *PW* does not strongly induce the Cyp1a detoxification system neither in the liver nor in the intestine, and non-metabolised naphthalene was detected in relative high levels in the liver, while the corresponding metabolites were not found in the bile in significantly higher levels over control. Fish exposed to 3-ring PAHs (*Oil* group) strongly induced the Cyp1a system both in the liver and intestine and high levels of bile metabolites were found. The heavy *PAH* exposure also induced Cyp1a in both liver and intestine, but bile metabolites where only observed in the early phase of the exposure.

Previous research in DNA adducts in fish has been performed with a similar study design using Northern pike (*Esox lucius*) [41, 105]. Increases in intestinal DNA adducts were observed after multiple oral doses (6 mg/kg fish) of single compounds or mixtures of three 5-ring PAHs: BAP, BKF and 7H-dibenzo[c,g]carbazole (DBC). Fish were exposed to multiple doses over weeks, and the fish were followed for more weeks for recovery after end of exposure [41]. DNA adducts were measured in liver, gills, brain and intestine. Rapid uptake was observed, and DNA adducts were detected in the liver one day after the first dose and in the intestine after three days. Levels of DNA adducts after multiple doses were 3 times higher in the intestine (347±17 nmol adduct/mol normal DNA) compared with the liver (110±9 nmol adduct/mol normal DNA). The clearance rate of DNA adducts was slow in the intestine, and no clearance was observed in the liver. Like the present study, these results also demonstrate that large parts of the metabolism of 5-ring PAHs are located in the intestine. Moreover, DNA adducts in fish are detectable soon after exposure, and they remain so even after many weeks of recovery.

The present study investigated effects of PAHs after oral and intraperitoneal exposure routes. Contribution from water exposure of PAHs may also affect gills and liver, and more data including the analysis of gills together with liver and intestine could improve the understanding of how different exposure routes affect different fish tissues.

## 5. Conclusion and recommendations

The present investigation documented that juvenile haddock responded quickly to both intraperitoneal injection and oral exposure of PAHs. High amounts of DNA adducts were detected in the liver 3 days after a single exposure dose. The difference in DNA adduct responses according to route of exposure should be considered in future monitoring programs, and we suggest that DNA adducts measurement should be performed in different tissues including liver, intestine and gills.

From the ^32^P-postlabelling assay, it was not possible to distinguish between the DNA adducts from the three different PAH sources, and the present study emphasizes the need of developing new MS based methods for identification of the DNA adducts generated from oil related pollution.

In Norway, the environmental objective concerning discharges from the O&G sector is that there should be zero harmful effects of discharges. When increased levels of DNA adducts in wild fish from areas with extensive O&G activities are reported, more effort should be used to understand which sources are contributing most to give advice for good management of the marine environment.

## Supporting information

S1 File (DOCX)

S2 File (DOCX)
