# Supplementary material for: DNA damage and health effects in juvenile haddock (Melanogrammus aeglefinus) exposed to PAHs associated with oil-polluted sediment or produced water
Source: PLoS One. 2020 Oct 22;15(10):e0240307. doi: 10.1371/journal.pone.0240307 (PMC7580938; doi:10.1371/journal.pone.0240307)
Supplement: S1 File — (DOCX) [file pone.0240307.s001.docx]

**DNA damage and health effects in juvenile haddock** (*Melanogrammus aeglefinus*) **exposed to PAHs associated with oil-polluted sediment or produced water**

**Supplementary data**

Sonnich Meier^1*^, Ørjan Karlsen^1^, Jeremie Le Goff^2^, Lisbet Sørensen^1,3^, Elin Sørhus^1^, Daniela M. Pampanin^4,5^, Carey E. Donald^1^, Per Gunnar Fjelldal^1^, Evgenia Dunaevskaya^5^, Marta Romano^6^, Ilaria Caliani^6^, Silvia Casini^6^, André S. Bogevik^7^, Pål A. Olsvik^1,8^, Mark Myers^9^, Bjørn Einar Grøsvik^1^.

^1^Institute of Marine Research, Box 1870, Nordnes, NO-5817 Bergen, Norway.

^2^ADn'tox, Bâtiment Recherche, Centre François Baclesse 3, Avenue du Général Harris, 14076 Caen Cedex 5, France

^3^SINTEF Ocean AS, Environment and New Resources, Trondheim, Norway

^4^Faculty of Science and Technology, Department of Chemistry Bioscience and Environmental Engineering, University of Stavanger, NO-4036 Stavanger, Norway

^5^NORCE, Mekjarvik 12, NO-4070 Randaberg, Norway

^6^Department of Physical, Earth and Environmental Sciences, University of Siena, via Mattioli, 4, 53100 Siena, Italy

^7^Nofima AS – Norwegian Institute of Food, Fisheries Aquaculture Research. Kjerreidviken 16, 5141 Fyllingsdalen, Norway

^8^Nord Univ, Fac Biosci & Aquaculture, Bodo, Norway

^9^Myers Ecotoxicology Services, LLC, USA

* Corresponding author Institute of Marine Research, Nordnesgaten 50, **5005 Bergen**, Norway.

E-mail address: sonnich.meier@hi.no

## S1. Background information for selection of PAH composition of the experimental feed.

The objective of this project has been to study the formation of DNA damage in haddock exposed to different petrogenic or pyrogenic polyaromatic hydrocarbons (PAHs). The main goal is to identify the sources of contaminants responsible for the DNA adducts observed in wild haddock caught around oil fields in the North Sea. Table S1 shows the PAH composition of produced water (PW) and sediment collected from Statfjord A, a North Sea oil platform at Tampen (Norwegian sector) (unpublished data supplied from Statoil). Background levels of PAHs in the North Sea were found in Boitsov et al. [1].

**Table S1.** PAH composition (%) and concentration (mg/L or mg/kg) in produced water from Statfjord A and sediments the from the same North Sea oil platform. The old drill cuttings were collected either just under the platform or in periphery of the deposited drill cuttings (unpublished data from Statoil). The marine sediment from Skagerrak shows the maximum background levels of PAH in the North Sea [1].

North Sea.

Clear differences in PAH composition have been identified, PW is dominated by 2 ringed PAHs (>90 %) with a small fraction of 3 ringed PAHs, but only very low amounts of heavy PAHs (≥4 ringed PAH). The drilling mud sediments also contain high levels of 2 ringed PAHs (44-62 %),but have in addition high amount of 3 ringed PAHs (26-50 %) and some heavy PAHs (≥4 ringed PAH). The background sediments are dominated by heavy PAHs (≥4 ringed PAH).

Both PW and drilling mud sediments have high amount of 2 ringed PAH. However, we did not wish to have too large overlaps in PAH compositions between the different exposure groups. Therefore, we did not mimic the PAH profiles in the sediments exactly, but tried to make exposures that were representative for the dominating PAHs from the different sources:

- *PW*: 2 ringed PAHs.
- *Oil* containing sediment; 3 ringed PAHs
- *PAH* background sediments; 4-6 ringed PAHs

## S2. Experimental food and PAH measurement

The PW feed was made by adding 60 g of PW extracts and 6 g of oil distillation fraction (Fr1; 240-320 ˚C). From this mixture were 28 g was dissolved into fish oil to a total weight of 200 g. This oil was again mixed with the total amount of fish oil (975 g) that were used for the vacuum coating of the 13 kg of pellets.

The oil feed was made by adding 30 g of oil distillation fraction Fr2 (320-375 ˚C) + 36 g Fr3 (375-420 ˚C) + 40 mg with pyrene standard. From this mixture were 27 g dissolved into the fish oil*.*

The *PAH* feed was made by weigh in 12 heavy PAHs (4-6 rings) from 34-212 mg (Table S3) and first dissolved it in 50 g of acetone and thereby into 200 g fish oil. The acetone was used to assist solubility of the heavy PAHs. The Acetone will evaporate during the vacuum coating and will not be present in the feed.

The PAH amount and composition in the experimental food were measured by extraction of the feed pellets and GC-MS analysis (Table S4).

The *PW* and the *Oil* feed contain a large mixture of other oil compounds, the PAHs are only contributing to 1.1% (PW mixture) and 1.5 % (Oil mixture) of the weight. It was only the PAHs that have been measured by analytical chemistry. However, Fig. S2 shows the GC-FID chromatograms of the total hydrocarbon, and it clear that both mixtures contain large amounts of alkanes and other oil compounds, like Unresolved Complex Mixtures (UCMs)


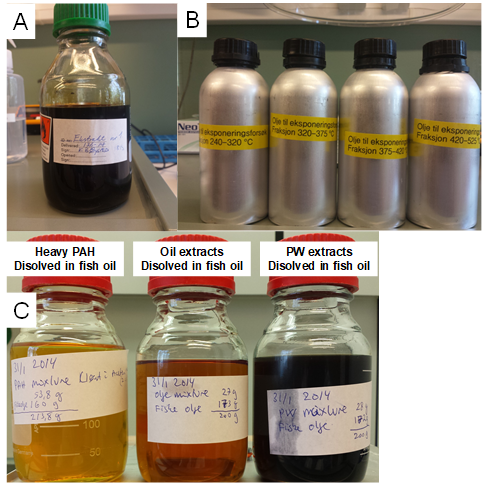


**Fig S1**. (A) Picture of produced water (PW) extract. (B) Pictures of the four oil distillation fraction that were available for the study. (C) Picture of the fish oil mixtures there were used to make the experimental food.

**Table S2.** Composition (mg) of the different oil fractions used to make the PW food (A) and the oil food (B). The PAH content was measured by GC-MS.

**Table S3.** Distribution of heavy PAHs in the “PAH feed”. Classification of carcinogenicity from the International Agency for Research on Cancer [2]. Group1: carcinogen to humans; Group 2A: probably carcinogen to humans; Group2B: possibly carcinogen to humans; Group3: not classifiable as to carcinogenicity to humans.

**Table S4.** Composition (mg/kg) of PAHs in the control feed and in the feed for the three PAH types of exposure. The feed pellets were extracted with *n*-hexane-DCM (1:1 v/v) and the PAH content was measured by GC-MS.

**Fig S2.** Gas chromatography analysis (GC-FID) of total hydrocarbons in A) produced water extract and B) Alkane standards (C12-C40), and oil distillation fraction (Fr1-4)

## S3. Autoradiograms


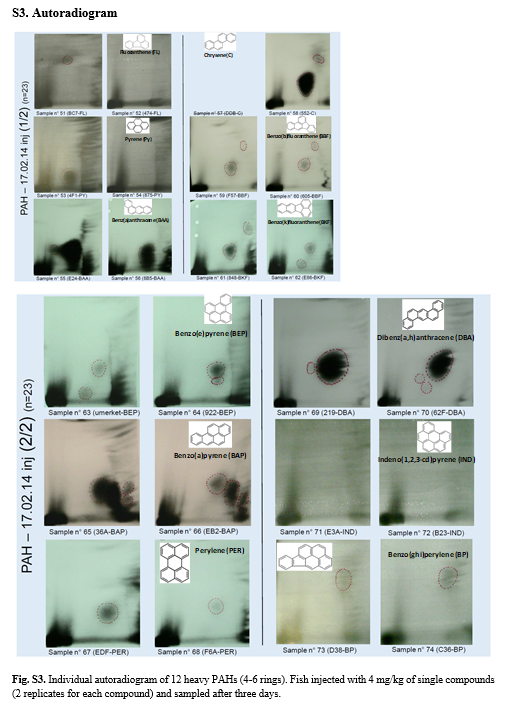


**Fig S3.** Individual autoradiograms of 12 heavy PAHs (4-6 rings). Fish injected with 4 mg/kg of single compounds (2 replicates for each compound) and sampled after three days.

## S4. Liver biomarkers: detection of gene expression, protein and enzyme activity

**Methods: *ahrr*, *gadd45a*, *gadd45a* and *p53* expression**

The total RNA was extracted from cod liver tissue with Promega Reliaprep simply RNA HT 384, art nr X9601(Nerliens) on a Biomek 4000 Laboratory Automated Workstation (Beckman Coulter) according to the manufacturer’s instructions and quantitated using a NanoDrop™-1000 spectrophotometer (Thermo Scientific). The RNA samples were normalised to the concentration of 100 ng/µL using the Biomek 4000 Laboratory Automated Workstation (Beckman Coulter). Reverse transcription was carried out using SuperScript® VILO™ cDNA Synthesis kit, art nr 11754050 (Life technologies) according to the manufacturer’s instructions, and the total RNA input was 500 ng in each reaction in a total volume of 10 µL.

PCR primers used to quantify the selected genes in cod were designed based on genome sequence for the respective species and shown in Table S5. The qPCR assay was run using Brilliant III Ultra-Fast SYBR® Green QPCR Master Mix, Catalog # 600882 (Life Technologies AS) according to the manufacturer’s instructions, with 2 µL of cDNA diluted 1 to 10 in a reaction mix containing 400 nM of forward primer, 400 nM of reverse primer in a total volume of 7 µl on a 384 well-plate. For the qPCR assay, amplification and fluorescence detection were performed by a QuantStudio™ 5 Real-Time PCR System (Applied Biosystems) for 40 cycles. Quality controls “no template controls” (ntc) and “no amplification controls” (nac) were run for quality assessment for each PCR assay.

Mean normalized expression (MNE) of the target genes was determined using a normalization factor based upon actb and uba52, calculated by the QuantStudio™ Design & Analysis Software.

**Table S5.** PCR primers, contig names and amplicon sizes.

| **Gene ID** | **Gene name** | **Marker for** | **Contig name** | **Forward primer** | **Reverse primer** | **Probe** | **Amplicon size (bp)** | **Assay type** |
| --- | --- | --- | --- | --- | --- | --- | --- | --- |
| *ahrr* | Aryl-hydrocarbon receptor repressor | Detoxification | >Soerhus-mRNA-2-dpf-1_CGATGT_L001_R1_001_(paired)_contig_92712 | AGCCAGACGCTGAACCTCAT | ATGCCGTGACCCTTGAACTC | n.a. | 122 | Sybr Green |
| *p53* | Tumor protein p53 | DNA damage | >Soerhus-mRNA-2-dpf-1_CGATGT_L001_R1_001_(paired)_contig_2079 | CCTGCTGAACTTCATGTGCAA | CCGAGAACATGCCCTTCAGA | n.a. | 102 | Sybr Green |
| *gadd45a* | Growth arrest and DNA-damage-inducible, alpha | DNA damage | >Soerhus-mRNA-2-dpf-1_CGATGT_L001_R1_001_(paired)_contig_13955 | ACGGTGTCAAAGGCAATCG | CTGGGTCCGCATTGAGAGAT | n.a. | 103 | Sybr Green |
| *gadd45g* | Growth arrest and DNA-damage-inducible, gamma | DNA damage | >Soerhus-mRNA-2-dpf-1_CGATGT_L001_R1_001_(paired)_contig_5185 | GTGCGCGTCAACGATATTGA | AAGGGTCTTTCCATGGGTTTG | n.a. | 121 | Sybr Green |
| *eef1a* | Eukaryotic translation elongation factor 1 alpha 1 | Reference gene | >Soerhus-mRNA-2-dpf-1_CGATGT_L001_R1_001_(paired)_contig_221 | CACATCGCCTGCAAGTTCAA | GGCTTGCTTGGGATCATGTT | n.a. | 128 | Sybr Green |
| *uba52* | Ubiquitin A-52 residue ribosomal protein fusion product 1 | Reference gene | >Soerhus-mRNA-2-dpf-1_CGATGT_L001_R1_001_(paired)_contig_5918 | TGAGGTCGAACCCAGTGACA | CTGCTTGCCAGCGAAGATC | n.a. | 103 | Sybr Green |
| *actb* | Beta actin | Reference gene | >Soerhus-mRNA-2-dpf-1_CGATGT_L001_R1_001_(paired)_contig_877 | ACAGCCGAGCGTGAGATTGT | TCGGGAAGCTCGTAGCTCTTC | n.a. | 125 | Sybr Green |
| *cyp1a* | Cytochrome P450, family 1, subfamily A | Detoxification | ENSGMOG00000000318 | CCTCCTTCCTGCCCTTCAC | TTGGGAATGAAGTAGCCATTGA | 6FAM-CCTCACTGCGCCACAAAAGACACATC-TAMRA | 83 | Taqman |
| *ef1α* | Eukaryotic translation_elongation factor_1 alpha 2 | Reference | ENSGMOG00000006122 | ATCGGCGGTATCGGAACA | GCTTGAGGACACCGGTCTCA | 6FAM-ACCCGTGGGCCGTG-MGB | 55 | Taqman |

**Fig S4.** Mean normalized expression (MNE) of *ahrr* (A), *p53* (B), g*add45g* (C) and *gadd45a* (D) by qPCR of the different treatments groups after two months of exposure. Data presented as average ± stdev. (*) indicate significant differences compared with control, p< 0.05.

**Fig S5.** Responses on CYP1A by ELISA with antibody towards CYP1A (A), effects on glutathione-S-transferase activity (GST) (B) and lipid peroxidation (LPO) (C) of the different treatments groups after two months exposure. Data presented as average ± stdev. For CYP1A detection we used polyclonal anti-trout CYP1A (CP-226, Biosense) diluted 1:1000. (*) indicate significant differences compared with control, p< 0.05. N = 10 individuals per group.

**Methods: Glutathione S-transferase (GST) activity and lipid peroxidation (LPO)**

Two additional biomarkers were measured in liver samples: GST activity is indicative of detoxification pathways, and LPO is a measure of oxidative stress. Samples were homogenized, using a Potter homogenizer, in phosphate buffer (0.1 M, pH 7.4) according to Ahmad et al. [3]. This homogenate was divided in two aliquots for GST and LPO analyse. The GST aliquot was further prepared by centrifuged at 13,400 g for 20 min (4°C), and the (postmitochondrial) supernatant was retained. The GST activity was determined in this supernatant following the conjugation of GSH with 1-chloro-2,4-dinitrobenzene (CDNB) by the method of Habig et al. [4] with some modifications. The reaction mixture consisted of 1.85 ml sodium phosphate buffer (0.1 M, pH 7.4), 0.050 ml reduced glutathione (1 mM), 0.050 ml CDNB (1 mM) and 0.050 ml PMS. Absorbance was recorded at 340 nm (25°C) and expressed as nmol CDNB conjugate formed/min/mg protein (ε = 9.6 x 10^3^ mM^-1^ cm ^-1^).

LPO levels were determined in the liver homogenate according to Ohkawa et al. [5] and Bird and Draper [6], with some modifications. Briefly, to 150 μl homogenate, 5 μl of 4% butylated hydroxytoluene (BHT) in methanol, was added. To this aliquot, 1 ml of 12% trichloroacetic acid (TCA) in aqueous solution, 0.90 ml Tris–HCl (60 mM, pH 7.4 and 0.1 mM DTPA) and 1 ml 0.73% 2-thiobarbituric acid (TBA) were added. The mixture was heated for 1 h in a water bath set at boiling temperature and then cooled to room temperature and centrifuged at 13,400 g for 5 min. Absorbance was measured at 535 nm and LPO expressed as nmol of thiobarbituric acid reactive substances (TBARS) formed/mg protein (ε = 1.56 x 10^5^ M^-1^ cm^-1^).

## S5. Liver damage

**
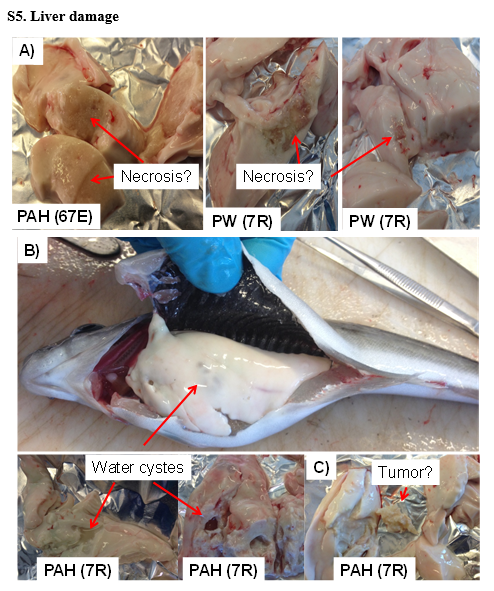
**

**Figure S6**. Macroscopically visible liver damage. At the end of exposure (67 days of exposure, 67E), 10 % (2 out of 20 fish) in the PAH group showed changes that appeared to be large areas of necrotic tissue. After one week of recovery (7R), the possible necrotic tissue was also seen in the PW group (2 out of 10 fish). In the PAH group, fish were found with large fluid-filled cysts (2 of 10 fish) and one fish with what appeared to be a solid nodule or tumor. However, this solid mass or nodule could not be confirmed by histological examination to be a true neoplasm.

**S6. Histopathology**

Liver samples were dissected, placed in histo-cassettes and stored into the histological fixative (3.7% formaldehyde) till embedding (4˚C). Samples were no thicker than 1 cm to ensure a proper fixation.

Histological sections (3 µm) were prepared at Helse Stavanger . Tissues were examined for health parameters related to physiological conditions, inflammatory and non-specific pathologies and those associated with pathogen and parasites infections.

Liver abnormalities were staged into mild, medium and severe, depending on the size of the tissue area affected in the sections and the degree of cellular change observed, as suggested by Lang, Wosniok (7), Benly et al. (2008) and Sensini et al. (2008). Each alteration was scored according to its severity and frequency (0 = absence of alteration, 1 = ≤ 10 % of the histological section showed the alteration, 2 = between 10% and 50% of the histological section showed the alteration, 3 = between 50% and 100% of the histological section showed the alteration).

The presence of parasites was scored as absent (0) or present (1). All micrographs were captured using an AxioCam MRc5 (Zeiss) digital camera mounted on a *Zeiss Axioplan 2* light microscope (Göttingen, Germany). All ssamples were analysed blind.

**Table S8 – Examples of histopathological alteration in haddock.**

| Histopathological alteration | Description |
| --- | --- |
| Small granulomas/melanomacrophage aggregates  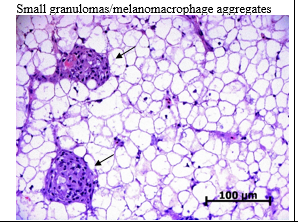 | Macrophage aggregates were present in haddock liver to varying degrees, either singly or in large numbers thorough liver parenchyma.  *167/A2 22.04*. |
| Granulomas  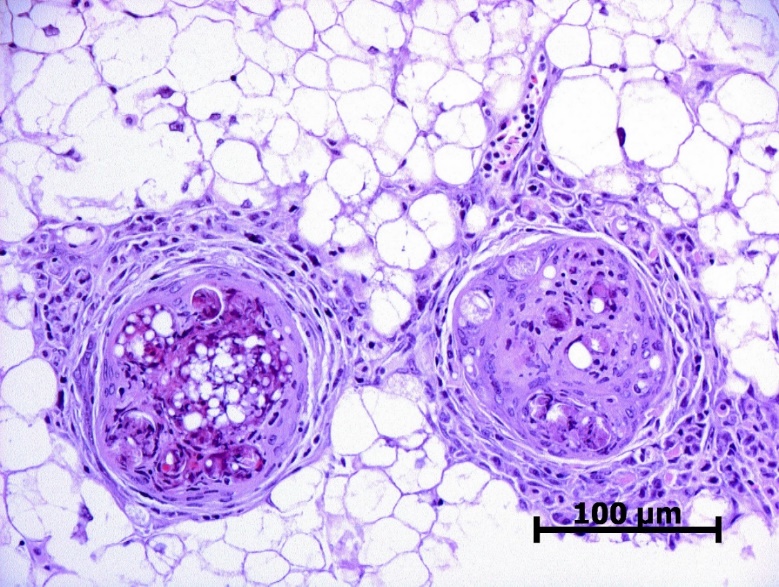  **CT**  **DT**  **L+MA** | Discrete capsule-like structures in the liver parenchyma. Early granulomas were characterized by a whorl of lymphocytes (L) and/or macrophages (MA) and by incomplete capsule of connective tissue (CT). Degenerative tissue (DT) is seen within the granuloma  *169. 22.04.* |
| Vacuolated focus  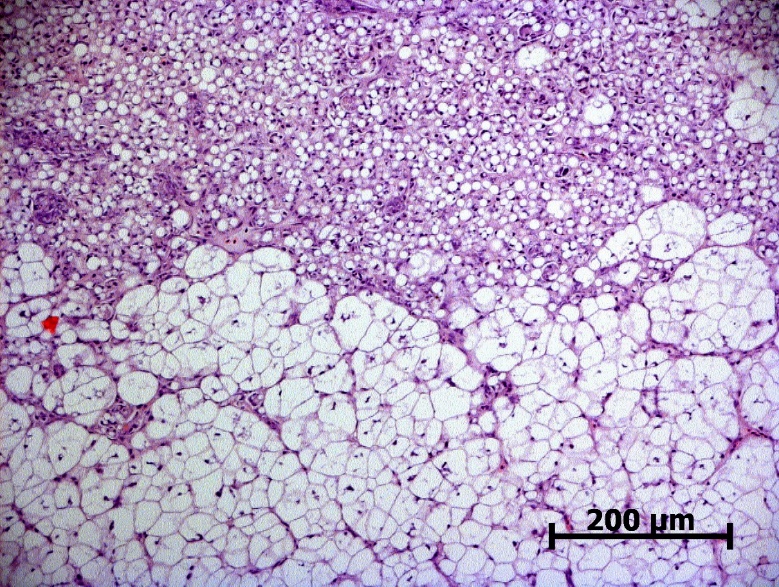  **Normal liver – high degree of vacuolation**  **Vacuolated focus** | The normal haddock liver is characterized by high degree of vacuolation. **Vacuolated focus** represents small hepatocytes with rounded vacuole. Could be part of the normal status of the liver.  *172/A7 22.04.* |
| Cirrhosis  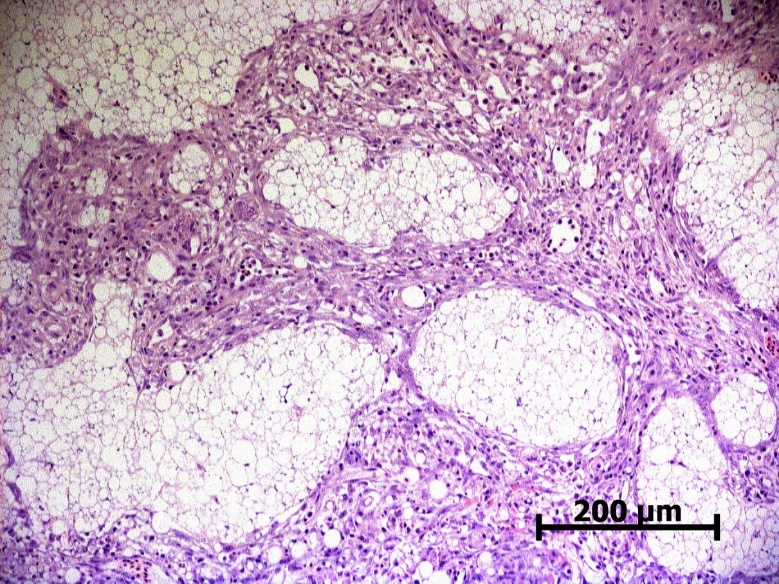  **Cirrhosis** | **Cirrhosis** (***fibrosis***) – proliferation of fibroblasts and accumulation of collagenous connective tissue. Characterized by presence of multi-layered connective tissue around granulomas, necrosis or affected tissue. Connective tissue forms walls separating nodules of hepatocytes.  *238 22.04.* |
| Complex pathology  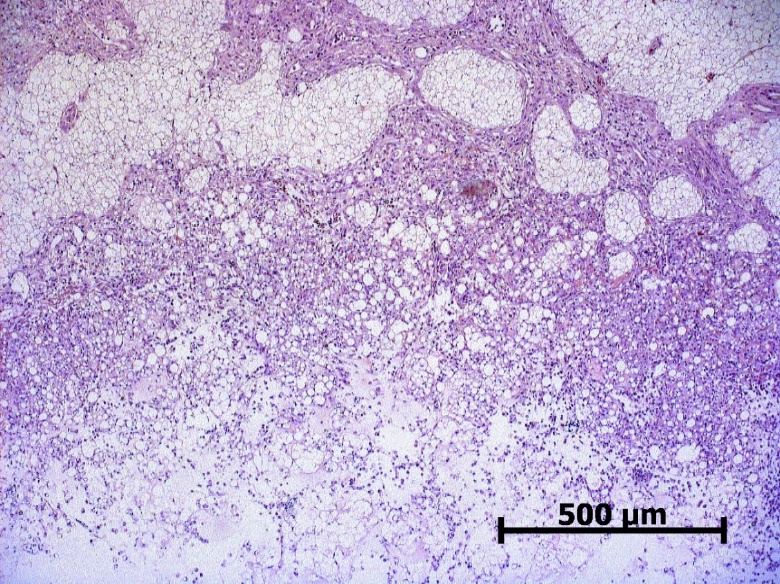  **Steatosis**  **Granulomatous inflammation**  **Necrosis**  **Cirrhosis** | **Necrosis** (cell death) – the cellular structure no longer maintained, eosinophilic cytoplasm elements and free pyknotic nuclei.  **Steatosis** (macrovesicular) – rounded hepatocytes contain a single enlarged vacuole.  *238/D13 22.04.* |
| Circulatory disturbances - Neoplastic blood cells  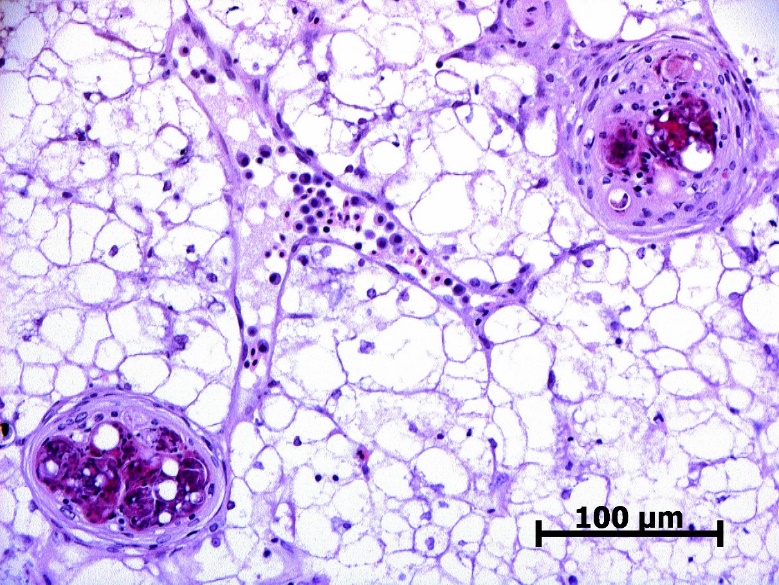 | Proliferation of abnormal blood cells (abnormal growth of some types of cells)  *255/A10 29.04.* |
| Circulatory disturbances – red blood cells ‘leakage’  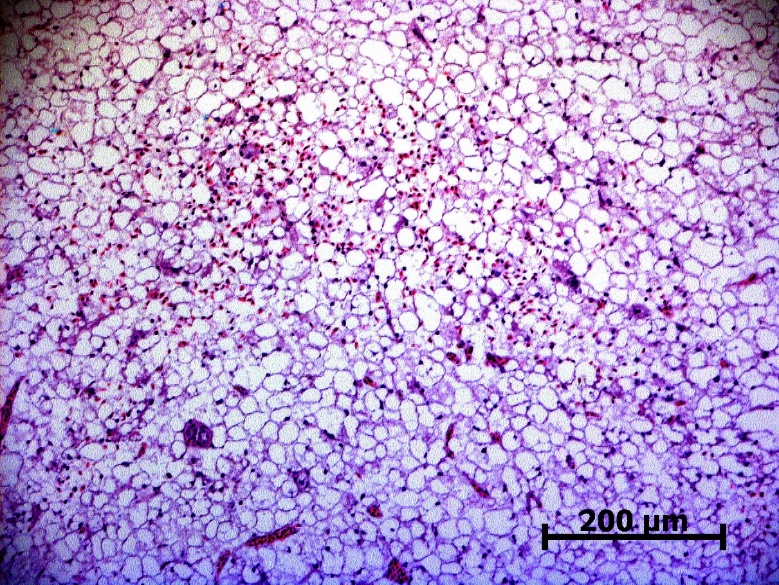 | ‘Leakage’ of blood cells from sinusoids (cause of injury?)  *P13 19.06* |
| Eosinophilic focus  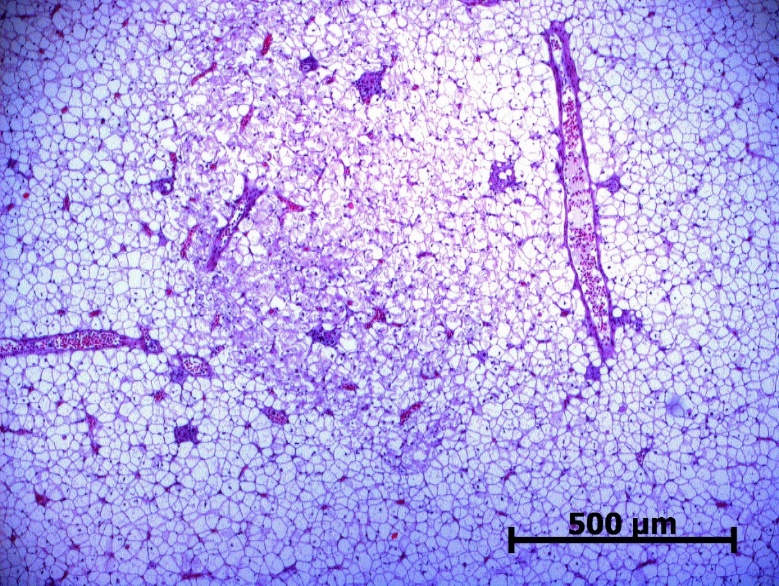 | Focus of cellular alteration appear as a discrete aggregations of hepatocytes, the morphology and staining characteristics of which differentiate them from the surrounding hepatocytes. Could be part of the normal status of the liver or may be induced by external factors.  *O32 19.06.* |

Reference List

1. Boitsov S, Petrova V, Jensen HKB, Kursheva A, Litvinenko I, Klungsoyr J. Sources of polycyclic aromatic hydrocarbons in marine sediments from southern and northern areas of the Norwegian continental shelf. Marine Environmental Research. 2013;87-88:73-84.

2. IARC. Some non-heterocyclic polycyclic aromatic hydrocarbons and some related exposures. 2010.

3. Ahmad I, Hamid T, Fatima M, Chand HS, Jain SK, Athar M, et al. Induction of hepatic antioxidants in freshwater catfish (*Channa punctatus* Bloch) is a biomarker of paper mill effluent exposure. BIOCHIMICA ET BIOPHYSICA ACTA-GENERAL SUBJECTS. 2000;1523(1):37-48.

4. Habig WH, Pabst MJ, Jakoby WB. Glutathione S-transferases. The first enzymatic step in mercapturic acid formation. Journal of Biological Chemistry. 1974;249(22):7130-9.

5. Ohkawa H, Ohishi N, Yagi K. Assay for lipid peroxides in animal tissues by thiobarbituric acid reaction. Analytical Biochemistry. 1979;95(2):351-8. doi: <https://doi.org/10.1016/0003-2697(79)90738-3>.

6. Bird RP, Draper HH. [35] Comparative studies on different methods of malonaldehyde determination. Methods in Enzymology. 105: Academic Press; 1984. p. 299-305.

7. Lang T, Wosniok W, Baršienė J, Broeg K, Kopecka J, Parkkonen J. Liver histopathology in Baltic flounder (*Platichthys flesus*) as indicator of biological effects of contaminants. Marine Pollution Bulletin. 2006;53(8–9):488-96. doi: <http://dx.doi.org/10.1016/j.marpolbul.2005.11.008>.
