# Supplementary material for: DNA damage and health effects in juvenile haddock (Melanogrammus aeglefinus) exposed to PAHs associated with oil-polluted sediment or produced water
Source: PLoS One. 2020 Oct 22;15(10):e0240307. doi: 10.1371/journal.pone.0240307 (PMC7580938; doi:10.1371/journal.pone.0240307)
Supplement: S2 File — (DOCX) [file pone.0240307.s002.docx]

This data are also available as an Excel file (but I was not able to upload it in the submission)

Table S6. Overview of sampling date and number of fish sampled

Table S7. Data for first sampling (3 days exposure). Control and PW

Table S7 (continue) Data for first sampling (3 days exposure). Oil and PAH.

Table S8. Data for abdominal cavity injection of heavy PAHs (3 days exposure).

Table S9. Data for second sampling (10 days exposure). Control and PW

Table S9 (continue). Data for second sampling (10 days exposure). Oil and PAH

Table S10. Data for third sampling (37 days exposure). Control

Table S10 (continue). Data for third sampling (37 days exposure). PW

Table S10 (continue). Data for third sampling (37 days exposure). Oil

Table S10 (continue). Data for third sampling (37 days exposure). PAH

Table S11. Data for fourth sampling (67 days exposure). Control

Table S11 (continue). Data for fourth sampling (67 days exposure). Control

Table S11 (continue). Data for fourth sampling (67 days exposure). PW.

Table S11 (continue). Data for fourth sampling (67 days exposure). PW.

Table S11 (continue). Data for fourth sampling (67 days exposure). Oil.

Table S11 (continue). Data for fourth sampling (67 days exposure). Oil.

Table S11 (continue). Data for fourth sampling (67 days exposure). PAH.

Table S11 (continue). Data for fourth sampling (67 days exposure). PAH.

Table S12. Data for fifth sampling (7 days recovery). Control

Table S12 (continue). Data for fifth sampling (7 days recovery). PW.

Table S12 (continue). Data for fifth sampling (7 days recovery). Oil.

Table S12 (continue). Data for fifth sampling (7 days recovery). PAH.

Table S13. Data for sixth sampling (58 days recovery). Control.

Table S13 (continue). Data for sixth sampling (58 days recovery). PW.

Table S13 (continue). Data for sixth sampling (58 days recovery). Oil.

Table S13 (continue). Data for sixth sampling (58 days recovery). PAH.
